# Methanol feeding strategies for high-yield production of a collagen-based protein in *Komagataella phaffii*

**DOI:** 10.1007/s00253-025-13675-z

**Published:** 2025-12-24

**Authors:** Jan Peter Ebbecke, Domenic Schlauch, Charlotte Güler, Hamidreza Pirmahboub, Selin Kara, Iliyana Pepelanova

**Affiliations:** 1 Institute of Technical Chemistry, Leibniz University Hannover, Hannover, Germany; 2 Cellbricks GmbH, Berlin, Germany

**Keywords:** Collagen, *Komagataella phaffii*, Recombinant protein expression, Bioprocess optimization, Methanol feeding strategy, Stirred tank reactor

## Abstract

**Abstract:**

The recombinant production of extracellular matrix proteins is a promising approach for replacing animal-derived materials in biomedical applications. *K. phaffii* represents a favorable expression host because it combines the ability of higher eukaryotes for secreted protein production with the ability to grow to high cell densities on simple, low-cost media. Additionally, this well-studied host allows for tight control of recombinant protein expression using the methanol-inducible AOX1 promoter. In this study, different methanol feeding strategies were evaluated to optimize the expression of a collagen-mimetic protein (ColMP-His). A methanol feed approach with carbon as a limiting nutrient resulted in the highest target protein production, whereas exponential feeding resulted in fast biomass accumulation with reduced protein expression. Moreover, the limited feeding strategy resulted in 25% lower oxygen consumption, despite the longer fermentation time, which has a positive impact on process cost efficiency. The application of a three-phases fermentation strategy with the addition of a preceding glycerol-fed batch phase to increase biomass did not improve product titers and was associated with reduced expression efficiency. A variation in the methanol feeding rate was also investigated for induction. A gradient-based methanol feed, which increased incrementally over time, achieved the highest final product concentration and sustained expression over extended fermentation periods. Compared with the initial process, the yield was increased by a factor of 11. Despite statistical limitations due to high variability, the results highlight the importance of adaptive process control in balancing cell growth and recombinant protein production. The presented gradient-based strategy provides a foundation for animal-free, scalable production of recombinant collagen materials.

**Key points:**

• *Methanol-limiting feed enhances collagen expression in Komagataella phaffii bioprocesses.*

• *Exponential feeding favors biomass but lowers protein yield and process efficiency*

• *Gradient feeding results in the highest collagen titers and sustained expression*

**Graphical abstract:**

**Figure:**
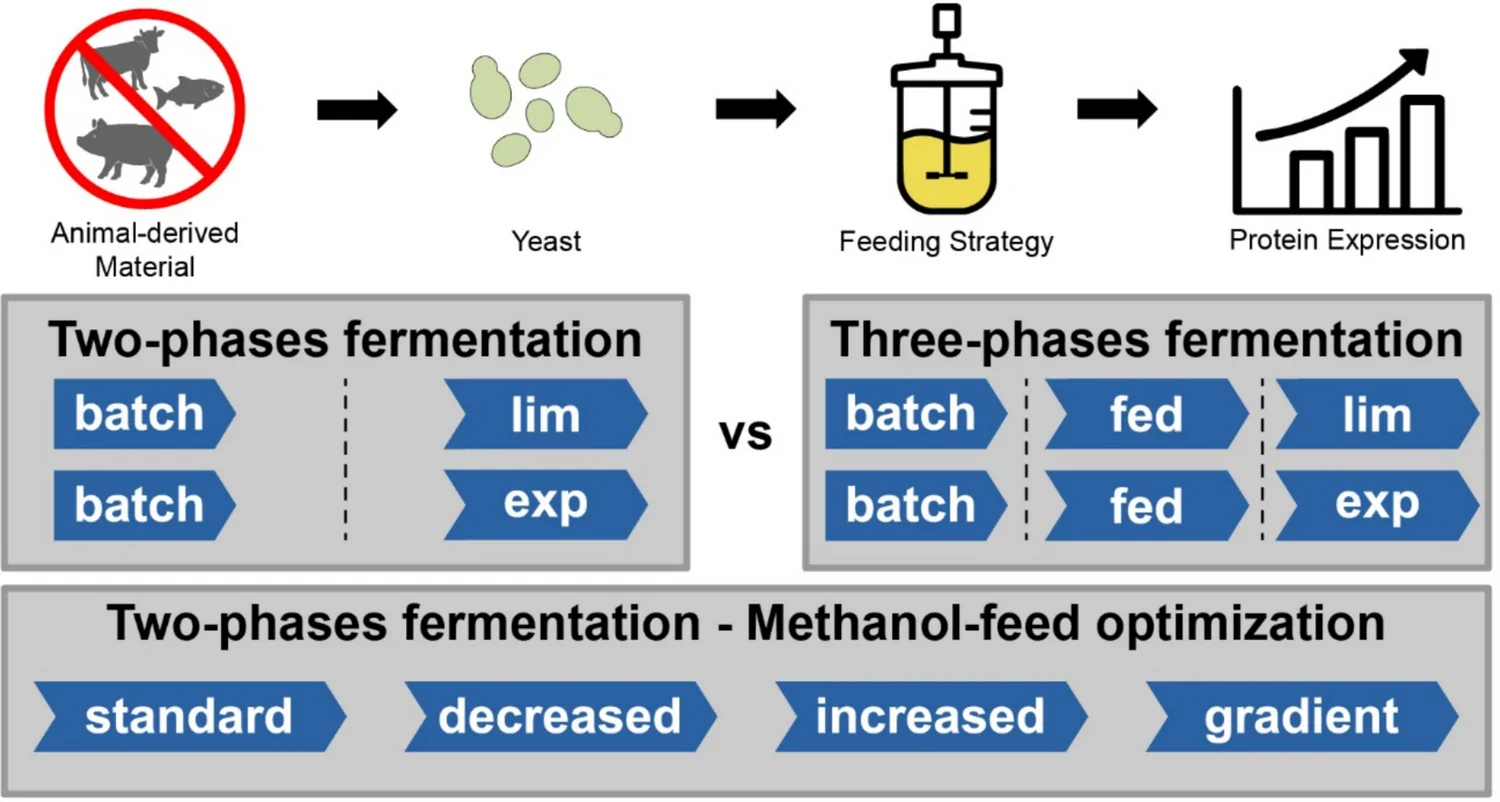

**Supplementary Information:**

The online version contains supplementary material available at 10.1007/s00253-025-13675-z.

## Introduction

Owing to their consistent quality, absence of animal-derived contaminants, defined molecular composition, and tunable mechanical and biological properties, recombinant collagens are increasingly recognized as valuable biomaterials for tissue engineering and regenerative medicine (Moura Campos et al. 2025). Unlike native collagen extracted from animal tissues, which suffers from batch-to-batch variability, immunogenic potential, transmission of pathogens, and ethical concerns, recombinant collagen offers improved safety, reproducibility, and design flexibility (Friess 1998). As such, recombinant collagen has been explored for a variety of biomedical applications, including tissue regeneration, skin substitutes, cartilage reconstruction, and bone grafts (Chen et al. 2020, 2023; Haagdorens et al. 2022; Cao et al. 2024).

Several groups have successfully produced recombinant collagens using different expression systems. Human collagen or collagen-like proteins have been recombinantly expressed in mammalian cell lines (Wang et al. 2024; Geddis and Prockop 1993; Fukuda et al. 1997), insect cells (Nokelainen 2000; Qi et al. 2016; Myllyharju et al. 1997), plants (Shoseyov et al. 2014; Stein et al. 2009; Xu et al. 2011), bacteria (Merrett et al. 2021; Xie et al. 2023; Rutschmann et al. 2014), and yeast (Nokelainen 2000; Ma et al. 2014, 2022; Williams and Olsen 2021; Myllyharju et al. 2000). Among these, *Komagataella phaffii* (*K. phaffii*) is considered particularly advantageous for scalable production because of its rapid growth, ability to reach high cell densities, cost-effective media requirements, and strong inducible promoters such as AOX1 (Cregg et al. 2000; Karbalaei et al. 2020). Furthermore, it allows for secretory expression, facilitating the downstream processing of complex proteins. Despite these advantages, the reported product yields for recombinant collagen are still insufficient for broader technical application in material fabrication.

We recently reported the design and characterization of a human-like collagen mimetic protein (ColMP-His), that was recombinantly produced in *K.* *phaffii*. This protein, which lacks proline hydroxylation, does not display thermal gelation under operating conditions, maintains high biocompatibility, and was shown to be photocrosslinkable for light-based 3D bioprinting applications (Schlauch et al. 2024). These properties make ColMP-His a promising candidate for replacing GelMA in stereolithographic bioinks. However, the major limitation of ColMP-His and other recombinant collagen-like materials is the low production titer in fermentation processes. To meet the practical demands of tissue fabrication, gram-scale quantities of purified recombinant protein are needed for experimental and clinical-scale 3D bioprinting. Based on typical bioink formulations (5–10% w/v), a single printing process, e.g., for a small tissue construct with a volume of 10 cm^3^ may consume several milligrams to grams of recombinant material. Achieving this level of production requires a significant improvement in process performance beyond previously reported yields of recombinant collagens (<0.6 g L^−1^) (Nokelainen et al. 2001).

To address this challenge, we aimed to increase the yield of recombinant collagen by controlling the bioprocessing conditions. A typical cultivation of *K.* *phaffii* involves a glycerol-based biomass growth phase followed by a methanol induction phase for recombinant protein expression. Several studies have demonstrated that recombinant protein productivity is highly sensitive to the feeding strategy and adaptation dynamics during methanol induction (Cregg et al. 2000; De et al. 2021). Key strategies include exponential methanol feeding based on online or offline methanol measurements for a constant methanol concentration in the media to prevent carbon source limitations and promote rapid growth (Chiruvolu et al. 1997; Jia et al. 2017; Gellermann et al. 2019), model-based exponential methanol feeding to maintain a constant specific growth rate (Celik et al. 2010, 2009; Boojari et al. 2023), constant methanol-limited feeding to favor protein production over biomass (Liu et al. 2011; Moser et al. 2017; Werten et al. 2001), batchwise or pulse feeding of methanol (Celik et al. 2007; Xiang et al. 2022), and implementation of a glycerol fed-batch phase to increase starting biomass before induction (Celik et al. 2007; Brierley 1998; Stratton et al. 1998). These strategies influence cell physiology, metabolic burden, and the balance between growth and expression. However, a systematic comparison of these feeding strategies for producing collagen-mimetic proteins is currently lacking in the literature.

We hypothesize that the expression of ColMP-His can be significantly enhanced by optimizing the methanol feeding strategy, particularly by dynamically adapting feed rates to match metabolic capacity during the induction phase. Furthermore, we investigated whether an increased starting biomass, achieved by a preceding glycerol fed-batch phase, can lead to higher product yields or whether it imposes limitations owing to oxygen demand or stress responses.

The aim of this study was to identify process parameters that optimize ColMP-His production in *K.* *phaffii*. We systematically compare exponential and limited methanol feeding strategies, assess the impact of additional biomass accumulation, and investigate various methanol feed rates during the induction phase. The effects on cell growth and protein expression are evaluated to derive a foundation for process scale-up and material provision for tissue engineering applications.

## Materials and methods

All chemicals were purchased from Carl Roth unless stated otherwise.

### Recombinant expression strain

The protein expression strain is based on previous work, and the molecular cloning has been described in detail elsewhere (Schlauch et al. 2024). In brief, a DNA sequence encoding a 59 kDa fragment of the human collagen I alpha 1 protein was inserted downstream of the alpha secretion signal in the pPIC9K vector (Invitrogen) under the control of the AOX1 promoter. A C-terminal His-tag was added to the sequence via PCR to generate the pPIC9K-ColMP-His plasmid. *K. phaffii* GS115 (Invitrogen) was transformed with the pPIC9K-ColMP-His plasmid via electroporation. Transformants were selected on histidine-deficient minimal dextrose media at 30 °C for 3 days. A selection of colony-forming units was screened in YPD media for positive ColMP-His expression. The expression strain with the best performance was selected for the experiments in this study.

### Preculture and seed culture

A preculture was prepared by inoculating 50 mL YPD media (10 g L^−1^ yeast extract, 20 g L^−1^ peptone, 10 g L^−1^ glucose (VWR)) with 500 µl from a frozen glycerol stock in a shake flask and incubated at 30 °C and 150 rpm overnight. A seed culture for the main fermentation was then prepared by inoculating 250 mL of YPD media with 1 mL of the preculture and again incubated at 30 °C and 150 rpm overnight.

### Bioreactor cultivations — exponential vs. limited methanol feeding strategy

The protein expression process was based on previous work (Gellermann et al. 2019; Schlauch et al. 2024). The main fermentations were conducted in a 2 L Biostat A glass reactor system (Sartorius, Germany). For the cultures, 1 L fermentation media (60 g L^−1^ glycerol, 0.9 g L^−1^ calcium sulfate dihydrate [VWR], 14.67 g L^−1^ potassium sulfate, 11.67 g L^−1^ magnesium sulfate heptahydrate [Merck], 9 g L^−1^ ammonium sulfate, 25.05 g L^−1^ sodium hexametaphosphate [Sigma–Aldrich], 200 µL L^−1^ Tego KS911 antifoam [Evonik, Germany], and 3.35 mL L^−1^ PTM1 trace salt solution [VWR]) was inoculated to an OD600 of 0.5 from the seed culture. During the entire fermentation process, the pH was constantly set to 5.0 with 3 M HCl and 25% ammonium solution, and the dissolved oxygen (DO) value was maintained at 30% saturation by controlling the stirring speed and providing supplemental oxygen to the ingas. The steady airflow of the ingas was 1.5 L air min^−1^ per liter of the initial fermentation volume. The end of the batch phase was reached after the complete consumption of glycerol, which was observed as a spike in the DO signal.

First, an exponential feeding strategy based on online methanol concentration measurements was compared to a methanol feed with a constant flow rate in a two-phase fermentation strategy (Table 1).

**Table 1:** Overview of exponential (1) and limited (2) methanol feeding strategies performed in a 2 L Biostat A glass reactor system (Sartorius, Germany). The fermentation phases are presented as separate growth and induction phases, including their respective durations. This represents the applied two-phases fermentation strategy

Methanol feeding strategy	Growth phase (Glycerol)	Induction phase (Methanol)	
Exponential (1)	Batch ~ 24 h	-	Constant concentration ~ 36 h
Limited (2)	Batch ~ 24 h	Adaptation ~ 5 h	Constant feed ~ 90 h

#### Methanol exponential strategy

For ColMP-His expression using the exponential methanol feeding strategy, a methanol sensor (Raven Inc. Biotech, Canada) was installed into the described reactor system. The sensor controlled a peristaltic pump via custom-made control software, which delivered methanol into the fermentation media of the reactor. To initiate the induction after the end of the growth phase, a methanol feed solution (methanol with 12 mL L^−1^ PTM1 trace salt solution) was manually added to the reactor to reach a concentration of 0.5% (v/v). The methanol concentration was maintained at a constant level of 0.5% by automatically running a pump once the methanol sensor values decreased below a preset value recorded during the initial methanol addition. The fermentation was terminated after 24 and 34,5 h of induction, respectively, because of reactor capacity limitations.

#### Methanol limited strategy

For the methanol-limited feeding strategy, the methanol feed solution (the “Methanol exponential strategy” section) was added to the reactor after the end of the growth phase via a methanol-calibrated peristaltic pump to ensure precise flow rates during fermentation. The feed rate was increased stepwise starting at 3.6 mL h^−1^ per L initial fermentation volume for 3 h, followed by 7.3 mL h^−1^ per L for 2 h. Once the culture had fully adapted to methanol utilization, the feed rate was increased to 10.9 mL h^−1^ per L and maintained throughout the remainder of the fermentation after 90 h of induction.

#### Three-phases fermentation strategy with additional glycerol feed

Furthermore, the impact of a three-phase fermentation strategy with an additional glycerol fed-batch growth phase was investigated (Table 2). Glycerol fed-batches were started after complete consumption of glycerol following the batch phase. Sterilized glycerol was added to the fermentation media using a glycerol-calibrated peristaltic pump. The feed rate was increased stepwise from 0.3 mL min⁻^1^ to 0.5 mL min^−1^ for 6 h in total. The methanol feed (the “Methanol exponential strategy” or "Methanol limited strategy” section) to initiate induction was started after complete consumption of glycerol from the fed-batch phase, again observed by a spike in the DO signal.

**Table 2:** Overview of exponential (1) and limited (2) methanol feeding strategies with an additional glycerol fed-batch growth phase performed in a 2 L Biostat A glass reactor system (Sartorius, Germany). The fermentation phases are presented as separate growth and induction phases, including their respective durations. This represents the applied three-phases fermentation strategy

Methanol feeding strategy	Growth phase (Glycerol)		Induction phase (Methanol)	
Exponential (1)	Batch ~ 24 h	Fed-batch ~ 6 h	-	Constant concentration ~ 24 h
Limited (2)	Batch ~ 24 h	Fed-batch ~ 6 h	Adaptation ~ 5 h	Constant feed ~ 90 h

### Bioreactor cultivations — optimization of a limited methanol feeding strategy

These fermentations were performed in a DASGIP® multibioreactor system (Eppendorf, Germany) equipped with 4 vessels of 1.8 L capacity each. For the main fermentation, each vessel was filled with an initial fermentation media volume of 0.7 L and run with the parameters described in the “Bioreactor cultivations exponential vs. limited methanol feeding strategy” section in a two-phases fermentation strategy. To optimize the limited feeding strategy, various methanol flow rates were investigated during induction (Table 3).

**Table 3:** Overview of the methanol flow rates used during the induction phase to optimize the limited methanol feeding strategy run in a DASGIP® multibioreactor system (Eppendorf, Germany). This represents the applied two-phases fermentation strategy

Limited methanol feeding strategy	Induction phase Methanol flow [mL h^−1^ L^−1^] initial fermentation volume	
	Adaptation	Methanol feed rate
Standard	3.6/7.3 ~ 5 h	10.9 ~ 120 h
Decreased	3.6/7.3 ~ 5 h	7.3 ~ 120 h
Increased	3.6/7.3 ~ 5 h	12.0 ~ 120 h
Gradient-based	3.6/7.3 ~ 5 h	10.9 + 1 (every 12 h) ~ 120 h

The methanol feed was started after complete consumption of glycerol from the batch phase, which was observed by a spike in the DO signal. Methanol feed solution (the “Methanol exponential strategy” section) was added to the reactor vessel via a methanol-calibrated peristaltic pump. For methanol adaptation, the feed rate increased stepwise, starting with 3.6 mL h^−1^ L^−1^ initial fermentation volume for 3 h, followed by 7.3 mL h ^−1^ L^−1^ for 2 h. Once the cultures had fully adapted to methanol utilization, four different feed rates were set. One feed rate was as described previously and defined as the standard (10.9 mL h^−1^ L^−1^), followed by a decreased feed rate (7.3 mL h^−1^ L^−1^) and an increased feed rate (12.0 mL h^−1^ L^−1^). These feed rates were maintained throughout the remainder of the fermentation process. Additionally, the fourth feed rate was increased in a linear stepwise manner throughout fermentation, starting at 10.9 mL h⁻^1^ L⁻^1^ and increasing by 1 mL h⁻^1^ L⁻^1^ every 12 h. This rate was referred to as the gradient-based feed rate. All these fermentation approaches were terminated after approximately 120 h of induction.

### Quantification of target protein expression in the supernatant via His Tag ELISA Detection Assay

For quantification of the expressed target protein from the supernatant, a His-Tag ELISA Detection Kit (GenScript Biotech, USA) was used. The supplier’s instructions were followed for the procedure, with the following exception. For the determination of the ColMP-His concentration, a previously purified ColMP-His protein, as described by Schlauch et al. (), was used as a reference substance. The sample was collected from the reactor vessel and centrifuged at 13,000 rpm for 10 min at 15 °C. The supernatant containing the target protein was filtered through a 0.2 µm membrane filter and stored at −20 °C until further use. All the samples were thawed immediately before analysis. The determination was performed in duplicates.

### Determination of wet cell weight (WCW)

The sample was collected from the reactor vessel and centrifuged at 13,000 rpm for 10 min at 15 °C. The supernatant was decanted, and the wet cell mass was weighed immediately in triplicates.

## Results

### Impact of exponential and limited methanol feeding strategies

A 58 kDa fragment of the human alpha-1 collagen I chain fused to a His-tag was expressed in *K. phaffii* and secreted into fermentation media in a 2 L single-vessel bioreactor system with a two-phases fermentation strategy. To investigate the optimal conditions for efficient recombinant protein expression, two fermentations were conducted under identical process parameters, differing solely in the applied methanol feeding strategy. In the first approach, a limiting feed strategy with a constant methanol flow rate was realized. In contrast, the second fermentation utilized an exponential feeding strategy designed to maintain a constant methanol concentration within the culture media.

**Fig. 1:**
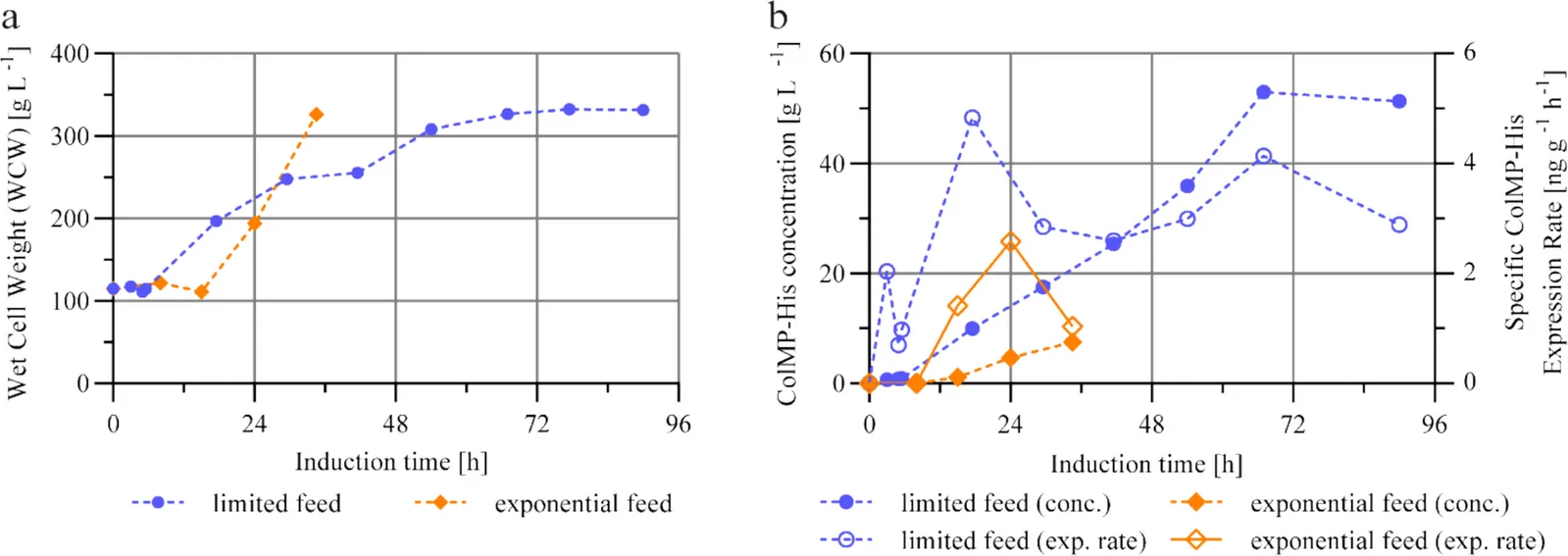
**a** Wet cell weight (WCW) [g L^−1^]. **b** ColMP-His concentration in culture supernatant [g L^−1^] (filled markers) and specific ColMP-His expression rate depending on WCW [ng g^−1^ h.^−1^] (unfilled markers) during the induction phase of two-phase fermentation strategy under two different methanol feeding strategies: limited feed (constant methanol flow into culture media) and exponential feed (constant methanol concentration in the culture media)

The cell growth, analyzed by wet cell weight (WCW), was evaluated (Fig. 1a) and showed different behaviors in these two fermentations, which can be attributed to their feeding strategies. Following the start of methanol addition, the limiting feeding strategy (blue) resulted in a delayed but continuous increase in wet cell weight, beginning 6 h after induction. This lag in growth initiation indicates a characteristic adaptation phase to methanol, as commonly reported in the context of the metabolic transition to C1 carbon sources (Dietzsch et al. 2011; Cai et al. 2021; Moser et al. 2017; Wang et al. 2023). Over the first 78 h of induction, the WCW increased from 100 g L^−1^ to 340 g L^−1^. Thereafter, cell growth plateaued, and the WCW remained stable until the end of the fermentation process. In comparison, the exponential feeding strategy (orange) did not result in a pronounced increase in the cell concentration during the initial hours following induction. A rapid increase in biomass was observed after 13 h, leading to a steep rise in WCW. Both fermentation strategies exhibited a common initial pattern. Immediately following the start of methanol addition, the cell concentration remained stagnant or slightly declined before entering a phase of sustained growth. This behavior further supports the presence of a methanol-induced metabolic transition phase, which appeared to be completed at approx. 6 h under limiting conditions and at 13 h under exponential feeding conditions. During the period from 13 to 36 h of induction, the cell concentration under the exponential feeding strategy increased from 100 g L^−1^ to 300 g L^−1^. Owing to the associated increase in culture volume and limitation by the reactor volumetric capacity, the fermentation process had to be terminated at this point.

Both strategies achieved a maximum specific growth rate (µₘₐₓ) of approx. 0.06 h⁻^1^ in the induction stage, albeit at different times. Under the limiting feeding strategy, µₘₐₓ was reached as early as 5 h to 5.5 h after induction, whereas under exponential feeding conditions, it occurred later, between 15 and 24 h. This observation highlights a shorter adaptation phase under limiting conditions, likely facilitated by the lower and more controlled methanol supply, which promoted earlier metabolic adjustment. While the limiting strategy actively constrained cell growth following adaptation, the exponential strategy, by maintaining a constant methanol concentration, enabled more rapid and intense biomass production within a shorter time frame. Growth rates throughout the entire processes are shown in Fig. S8a (supplementary information).

Protein secretion was analyzed by SDS-PAGE and is shown in Fig. S1 (supplementary information). Both methanol-feeding strategies based cultivations showed ColMP-His expression. A protein band with increasing intensity during the induction phase at an apparent molecular weight between 100 and 130 kDa was observed. The observed molecular weight of ColMP-His is approximately twice the theoretical size of the protein, which is expected to be 58.8 kDa. Similar observations have been reported for various recombinant collagen-derived proteins, which migrate at higher apparent molecular weights in SDS-PAGE (Werten et al. 2001, 1999; Gellermann et al. 2019; Butkowski et al. 1982). This aberrant migration behavior has been hypothesized to result from increased structural rigidity of the protein (Furthmayr and Timpl 1971), or from a reduced content of hydrophobic amino acid residues (Toshihiko Hayashi and Yutaka Nagai 1980). However, the presence of the recombinantly expressed ColMP-His protein was confirmed via Western Blot using an anti-His₆-tag antibody.

The protein content in the fermentation supernatant was determined via His-Tag ELISA Detection assay. The target protein concentration for the exponential (orange) and limited (blue) feeding strategies is shown in Fig. 1b. Under the limiting methanol feeding strategy, protein expression commenced 6 h after methanol addition and subsequently increased in correlation with rising cell density. Over the course of the first 66 h of induction, the concentration of ColMP-His in the cell-free culture supernatant increased to 53.0 g L^−1^, corresponding to an average expression rate of 0.9 g L^−1^ h^−1^. In a later phase of induction, a slight decrease in protein concentration was observed, resulting in a final ColMP-His titer of 51.3 g L^−1^ at the end of fermentation. In contrast, under the exponential feeding strategy, detectable ColMP-His expression was observed only after approx. 15 h of methanol induction. From that point onward, the protein concentration increased steadily, reaching a maximum of 7.5 g L^−1^ after 35 h, corresponding to an average expression rate of 0.4 g L^−1^ h^−1^. This value was markedly lower than that obtained under limiting conditions.

The difference in biomass-specific protein expression becomes particularly evident when comparing periods in which the cell densities in both fermentations were approximately equivalent. In an interval of the induction phase where the WCW levels were similar, the ColMP-His concentration under the limiting strategy increased from 10.0 to 53.0 g L^−1^, corresponding to an expression rate of 0.9 g L^−1^ h^−1^. Under the same conditions, the protein concentration with the exponential feeding strategy increased from 4.7 to 7.5 g L^−1^, resulting in an expression rate of 0.2 g L^−1^ h^−1^. These observations suggest a lower protein expression efficiency under exponential feeding, particularly in relation to the available biomass. The effects are illustrated in Fig. 1b, which depicts the specific ColMP-His expression rate as a function of WCW over the entire course of the induction phase.

The limiting methanol feeding strategy (blue) resulted in an early increase in the specific protein expression rate, which reached an initial maximum of 4.8 ng g^−1^ h^−1^ ColMP-His per WCW at 18 h after the start of induction. Subsequently, the expression rate declined to 2.6–3.0 ng g^−1^ h^−1^ between 30 and 54 h. Thereafter, a second increase was observed, reaching a second peak of 4.1 ng g^−1^ h^−1^ at 67 h. By the end of the induction phase, a pronounced decrease was detected, with the specific expression rate dropping to 2.9 ng g^−1^ h^−1^ at 90 h, which may indicate the degradation of previously accumulated protein, potentially caused by limiting factors or cellular stress.

The initially high expression activity suggests that the cells were metabolically active during the early phase of induction and capable of efficiently synthesizing the target protein. The subsequent decline in specific productivity may be attributed to physiological stress (Zahrl et al. 2017), metabolic reprogramming (Heyland et al. 2011), or depletion of intracellular resources (Garrigós-Martínez et al. 2021). The second increase at 67 h may indicate that the cells regained productivity following a period of reduced expression, possibly due to adaptive responses to changing environmental conditions. In contrast, the decline toward the end of fermentation likely reflects the beginning of limiting conditions, such as the accumulation of inhibitory metabolites (Jordà et al. 2012) or nutrient depletion.

In contrast, the exponential feeding strategy (orange) resulted in a delayed increase in the specific protein expression rate, which commenced 8 h after induction and reached a maximum of 2.6 ng g^−1^at 24 h. Over the subsequent 12 h, the rate decreased to 1.0 ng g^−1^. Further extension of the induction phase was not possible, as rapid biomass accumulation led to the reactor’s volume capacity being reached, necessitating early termination of the fermentation process.

Overall, the specific protein expression rate under exponential feeding conditions remained substantially lower than that observed with the limiting strategy. This finding indicates that although the cells proliferated more rapidly under the exponential methanol supply, they exhibited reduced efficiency in terms of recombinant protein production. The results suggest that the exponential strategy favors biomass accumulation, but compromises the productivity of the target protein, as reflected by the consistently lower specific expression rates.

### Impact of the three-phases fermentation strategy with additional glycerol fed-batch growth phase

To specifically investigate the influence of elevated biomass levels at the start of methanol-induced protein expression, a three-phases fermentation strategy with additional glycerol fed-batch was implemented immediately following the glycerol batch phase. Subsequent induction with methanol was then carried out using either a limiting or an exponential feeding strategy.

**Fig. 2:**
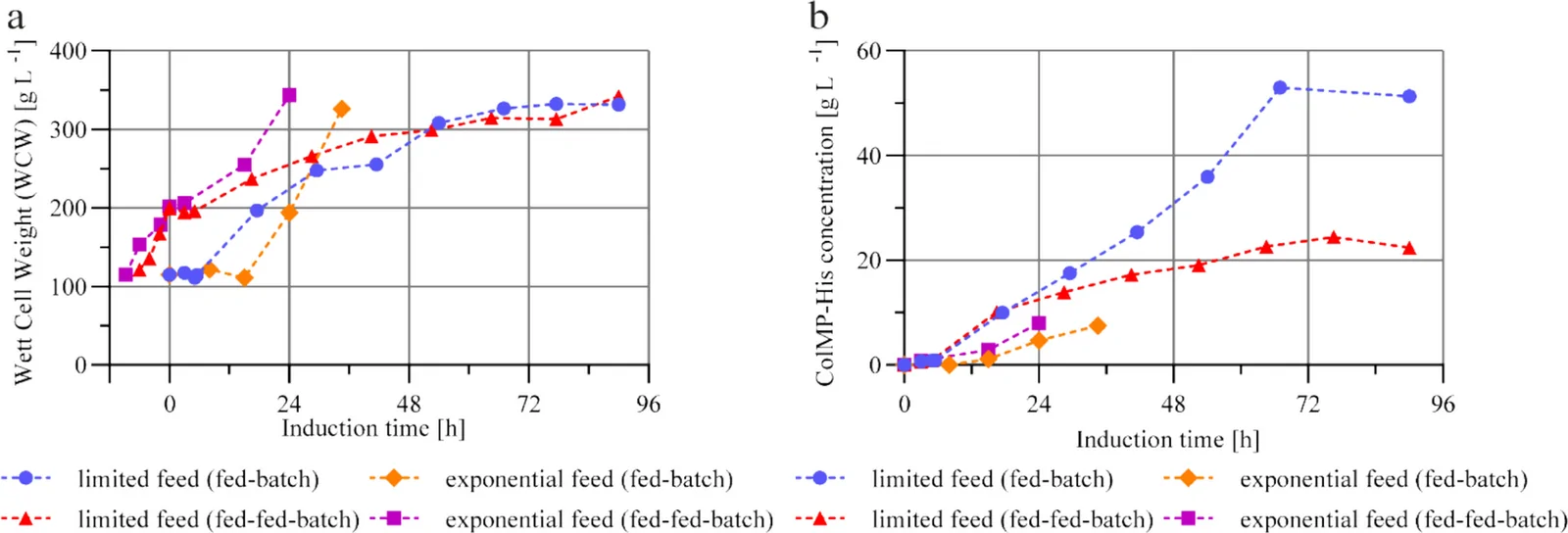
**a** Wet cell weight (WCW) [g L^−1^] during the induction phase of three-phase fermentation strategy with an additional glycerol fed-batch phase (fed-fed-batch, red/violet) and two-phase fermentation strategy without (fed-batch, blue/orange). Two different methanol feeding strategies were investigated: limited feed (constant methanol flow into the culture media) and exponential feed (constant methanol concentration in the culture media). **b** ColMP-His concentration in cell-free culture supernatant [g L.^−1^] during the induction phase of fermentation with an additional glycerol fed-batch phase (fed-fed-batch, red/violet) and without (fed-batch, blue/orange). Two different methanol feeding strategies were investigated: limited feed (constant methanol flow into the culture media) and exponential feed (constant methanol concentration in the culture media)

Biomass formation was assessed by monitoring the wet cell weight over the entire course of the induction phase, including the preceding glycerol fed–batch growth phase (Fig. 2a).

The effect of a preceding glycerol fed-batch phase on cell growth during the methanol-induced phase is shown in Fig. 2a. Here, the additional glycerol supplementation under the limiting feeding strategy (red) resulted in a higher initial biomass, with methanol feeding initiated at a WCW of 200 g L^−1^. In the absence of this phase (blue), induction began at a lower WCW of 115 g L^−1^. In both processes, a methanol adaptation phase of comparable duration was observed, followed by an increase in biomass 6 h after the start of methanol addition. In the range of 6 h to 24 h following methanol feeding, biomass formation was greater in fermentation conducted without the preceding glycerol fed-batch phase. This behavior may reflect a stronger impact of methanol-related limitations or increased cellular stress associated with the higher initial cell density in the variant that included the additional growth phase. The observed differences are further supported by the respective maximum specific growth rates. While a µₘₐₓ of 0.06 h⁻^1^ was reached in the absence of glycerol supplementation, the corresponding value under glycerol-fed conditions was substantially lower at 0.02 h⁻^1^. Growth rates throughout the entire processes are shown in Fig. S8 b (supplementary information). As the induction progressed, the cell concentrations of the glycerol batch and fed-batch strategies gradually converged. After 48 h, the WCW values showed minimal differences, and by the end of the induction phase, both processes reached a maximum WCW in the range of 330–350 g L^−1^.

Under exponential methanol feeding conditions, the initial WCW values at the start of induction were 200 g L^−1^ with the glycerol fed-batch phase (Fig. 2a, violet) and 115 g L^−1^ without (Fig. 2a, orange). In contrast to the limiting strategy, the methanol concentration in the medium was abruptly increased to 0.5% (v/v). It remained constant under exponential feeding compared with the constant feeding rate, resulting in methanol limitation. Higher initial cell densities appeared to tolerate this change in methanol concentration more effectively, as reflected by a shortened adaptation phase in the fermentation that included the additional glycerol phase. In this glycerol-fed variant, biomass accumulation resumed as early as 2 h after induction, whereas in the non-fed counterpart, this occurred after 14 h. Due to the higher initial WCW and the shortened adaptation phase, a final cell concentration of 340 g L^−1^ was achieved after 24 h of induction. However, further extension of the process was not feasible, as the increasing culture volume led to the reactor’s maximum working volume being reached. Furthermore, the oxygen transfer capacity as well as heat transfer capacity were reached. In fermentation without the glycerol phase, induction also had to be terminated after 36 h because the reactor reached its maximum working volume. At the time of termination, the WCW was 330 g L^−1^.

In addition to biomass formation, protein secretion into the culture supernatant was also investigated via SDS-PAGEs and the presence of the recombinantly expressed ColMP-His protein was previously confirmed via Western Blot. This SDS-PAGE analysis revealed positive ColMP—His expression with the glycerol batch and fed-batch strategies (Fig. S2). The progression of the ColMP-His concentration, determined by a His-Tag ELISA detection assay, in the cell-free culture supernatant during the induction phase of fermentation under limiting and exponential feeding strategies without (blue, orange) and with (red, violet) an additional glycerol fed-batch phase before methanol induction is shown in Fig. 2b. Following completion of the methanol adaptation phase, the ColMP-His concentration under the limiting feeding strategies increased at a similar rate in the range from 5 to 17 h in both setups, despite the differing initial cell densities. During the subsequent induction phase, from 17 to 65 h, a substantially greater increase in protein concentration was observed in the fermentation without the additional glycerol phase, reaching a maximum of 53.0 g L^−1^. In contrast, the glycerol-fed variant reached 22.6 g L^−1^ within the same timeframe, corresponding to 43% of the yield obtained in the process without a glycerol fed-batch. After 65 h of induction, both processes reached a stable or slightly declining plateau in protein concentration. This lower expression rate, despite the higher cell mass, might result from some sort of substrate limitation, as growth rates also declined in the glycerol fed-batch conditions within this time frame. Additionally, high biomass levels may have induced cellular stress, potentially leading to a reallocation of metabolic resources in favor of maintenance and growth, at the expense of recombinant protein production.

The analysis of the exponential methanol feeding strategy, shown in Fig. 2b, revealed an earlier increase in the ColMP-His concentration during fermentation with a preceding glycerol fed-batch phase (violet), which commenced as early as 3 h after induction. In contrast, a comparable increase in fermentation without an additional glycerol-fed phase (orange) was observed only after 15 h. This observation is consistent with the shortened methanol adaptation phase, which was likely facilitated by the higher initial biomass and the associated increase in cellular metabolic capacity. The earlier metabolic transition in the glycerol fed-batch variant resulted in an accelerated onset of recombinant protein expression. A final ColMP—His concentration of 8 g L^−1^ in the cell-free culture supernatant was reached after 24 h of induction. Moreover, the protein concentration in the fermentation without the glycerol fed-batch phase was 4.7 g L^−1^. This value continued to increase over the following 12 h, reaching a level comparable to that of the glycerol-fed process, before the fermentation had to be terminated owing to the reactor’s volume capacity being reached. In fermentation with glycerol supplementation, no observations beyond 24 h were possible, because this process also had to be terminated owing to volumetric limitations. Ultimately, these results demonstrate that the exponential feeding strategy, when combined with a glycerol fed-batch phase, enabled the achievement of a comparable final ColMP-His concentration within an induction period that was 12 h shorter. However, when considering the overall process duration, including the additional glycerol fed-batch phase, the advantage of the shortened induction time becomes less pronounced with respect to the total process-related product yield over time. A comprehensive summary of the process parameters and yields from the performed fermentations is shown in Tab. S1 (Supplementary information).

To assess cell-specific expression efficiency, specific protein expression was analyzed as a function of the corresponding wet cell weight (WCW) over the induction period. The fermentation without the additional glycerol fed-batch phase resulted in a consistently higher specific expression rate throughout the induction period (Fig. S4a). An initial maximum of approx. 4.8 ng g^−1^ h^−1^ ColMP-His per WCW was reached after 18 h. A second peak at approx. 65 h of induction suggests that cells can remain productive over extended periods. During fermentation with an additional glycerol fed-batch phase, a maximum specific expression rate of 3.7 ng g^−1^ h^−1^ was reached at 17 h. However, a pronounced decrease in the expression rate was observed thereafter, which may indicate metabolic burden or limited nutrient availability for the cells. Notably, the specific expression maximum in this variant occurred at the same time compared to fermentation without glycerol supplementation.

A similar pattern was observed under the exponential feeding strategy (Fig. S4b). In both fermentations, the maximum specific expression rate was reached within the first 24 h of induction. In the variant with an additional glycerol fed-batch phase, the specific rate increased at an earlier time point, whereas in the fermentation without glycerol supplementation, a rise was observed only after 8 h. In direct comparison with the limiting feeding strategy, fermentations conducted under exponential feeding conditions presented lower specific expression rates. The observed maximum values were 50% lower than those achieved under limiting conditions. This finding reinforces the previously noted tendency of exponential methanol feeding to primarily promote biomass accumulation, while compromising expression efficiency relative to the cell mass.

### Impact of methanol feed rate variation in the limited feed strategy

The influence of the methanol feed rate on protein expression was further investigated. For this purpose, recombinant ColMP-His was expressed under different methanol feeding rates. Based on the previously used methanol feed rate of 10.9 mL h⁻^1^ L⁻^1^ (relative to the initial culture volume), which was defined as a standard feed rate, three additional feed variants were evaluated: an increased feed rate of 14.5 mL h⁻^1^ L⁻^1^, a reduced feed rate of 7.3 mL h⁻^1^ L⁻^1^, and a stepwise-increasing gradient feed rate in which methanol addition was increased by 1 mL h⁻^1^ L⁻^1^ every 12 h, starting from the standard value of 10.9 mL h⁻^1^ L⁻^1^.

**Fig. 3:**
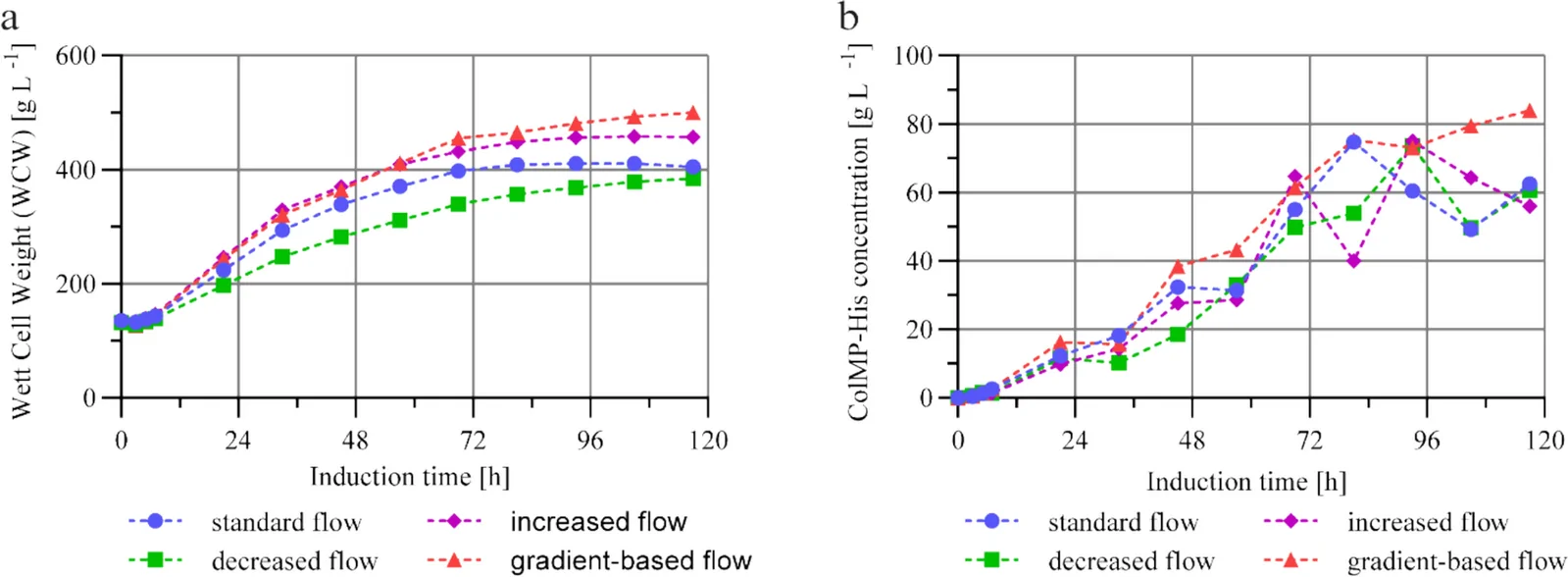
**a** Wet cell weight (WCW) [g L^−1^] and **b** ColMP-His concentrations in the cell-free culture supernatant [g L^−1^] during the induction phase of two-phase fermentation strategy processes with limited methanol feeding strategy and different methanol flow rates: standard flow (10.9 mL h^−1^ L^−1^_initial volume_), increased flow (14.5 mL h^−1^ L^−1^_initial volume_), decreased flow (7.3 mL h^−1^ L^−1^_initial volume_) and gradient-based flow (10.9 mL h^−1^ L^−1^_initial volume_ + 1 mL h^−1^ L.^−1^_initial volume_ every 12 h)

Cell growth was assessed by monitoring the WCW over the induction period (Fig. 3a). For all the tested methanol feed rates, continuous biomass accumulation was observed until 72 h to 96 h after induction. Beyond this point, the rate of cell growth decreased across all fermentations, which is characteristic of the transition into the stationary phase.

The fermentation with the reduced methanol feed rate resulted in the lowest overall biomass accumulation throughout the process. The maximum WCW reached approx. 384 g L^−1^ at the end of the induction phase. Slightly higher values were observed in the variant operated under the standard methanol feed rate, which reached a maximum WCW of approx. 411 g L^−1^ after 81 h. A slight decline in biomass was observed thereafter, indicating entry into the stationary phase, likely due to nutrient limitation.

Fermentations with an increased and gradient-based methanol feed rate resulted in comparatively accelerated cell growth with no substantial differences until approx. 57 h. However, fermentation with an increased methanol feed rate resulted in reduced growth compared to the gradient-based process after 69 h. This variant reached a maximum WCW of 456 g L^−1^ at 93 h, which remained stable thereafter, indicating entry into the stationary phase. In contrast, the gradient-based methanol feeding strategy resulted in continuous biomass accumulation throughout the entire observation period. No distinct stationary phase was observed, and the final WCW reached 500 g L^−1^ at the end of the induction phase, representing the highest biomass concentration among all fermentation conditions tested. These results suggest that, under these conditions, an extension of the induction phase might have led to a further increase in biomass.

The maximum specific growth rate (µₘₐₓ) in the induction phase was reached between 7 and 21 h across all the feeding strategies. An increase in the methanol feed rate was associated with a corresponding increase in µₘₐₓ, indicating that greater methanol availability supported more rapid biomass accumulation during the early induction phase (reduced: µₘₐₓ = 0.025 h⁻^1^, standard: µₘₐₓ = 0.032 h⁻^1^, increased: µₘₐₓ = 0.037 h⁻^1^, and gradient: µₘₐₓ = 0.037 h⁻^1^).

As expected, a low methanol feed rate limited cell growth, whereas a high feed rate, although potentially toxic, did not exhibit inhibitory effects in this study and was growth-promoting. Among the tested conditions, the gradient-based strategy proved to be the most effective approach for maximizing cell growth under controlled methanol induction. However, it is important to emphasize that high cell density does not necessarily correlate with increased protein expression. Given the focus of this study on maximizing recombinant protein yield, optimizing cell growth alone cannot be considered sufficient.

Target protein secretions, analyzed by SDS–PAGE, are shown in Fig. S3. All the fermentations presented positive ColMP—His expression, as previously confirmed by Western Blot. The protein content in the culture supernatant was again quantified via an ELISA targeting His-Tag proteins. All process variants presented a continuous increase in the ColMP-His concentration during the first 70 h of induction (Fig. 3b). However, in the subsequent phase, fluctuating values made a clear interpretation of the protein concentration difficult. One reason could be that each methanol feed rate condition was performed only once. However, a more important factor is the applied quantification method, which may have introduced measurement inaccuracies. At the time of investigation, this approach represented the only available option for target protein determination as described previously. Among all the variants, fermentation with the reduced methanol feed rate yielded the lowest ColMP-His concentrations throughout the process, peaking at 73.7 g L^−1^ after 93 h and decreasing to 60.6 g L^−1^ by the end of induction. The standard feed rate reached a maximum of 74.8 g L^−1^ at 81 h and decreased to a final concentration of 62.5 g L^−1^. Under increased methanol feed conditions, the protein concentration had the highest value of 74.9 g L^−1^ at 93 h, followed by a decrease to 56.0 g L^−1^ at the end of induction. In correlation with the biomass profiles, a general trend was observed. As soon as cell growth stagnated or transitioned into the stationary phase, a decrease in protein concentration occurred. This effect may be attributed to limited nutrient availability, caused by a restricted methanol supply and increasing cellular stress at high biomass densities. This potentially leads to increased protein degradation. The gradient-based strategy differed from the other fermentation procedures. No decline in protein concentration was observed in this variant. The ColMP-His levels continued to rise throughout the induction period, reaching a final concentration of 83.9 g L^−1^ after 120 h. Accordingly, this strategy yielded the highest biomass and the highest final protein concentration among all the tested methanol feeding conditions. A comprehensive summary of the process parameters and yields from the performed fermentations is presented in Tab. S2 (Supplementary information).

## Discussion

The comparison between limited and exponential methanol feeding strategies highlights fundamental differences in terms of cell growth and protein expression efficiency. While a limited methanol supply resulted in slower, yet over a wide range of continuous biomass production and protein expression, exponential feeding led to more rapid cell growth. However, this apparent advantage was offset by markedly lower specific protein productivity. Under limited conditions, the maximum specific expression rate reached 4.8 ng g⁻^1^ h^−1^ WCW, whereas under exponential feeding, 2.6 ng g⁻^1^ h^−1^ WCW was achieved. Accordingly, the methanol-limited strategy yielded a final ColMP-His concentration of 51.3 g L⁻^1^, which was more than seven times greater than that obtained with exponential feeding (7.5 g L⁻^1^). Moreover, the rapid increase in culture volume observed in the exponential setup led to early termination of the induction phase due to reactor volume limitations, thereby precluding sustained protein production over an extended fermentation period. The reduced expression of the target protein under exponential conditions is most likely attributable to increased metabolic burden. A study demonstrated that excessively high methanol feed rates can alter metabolic pathways and increase intracellular stress due to disturbance of the tricarboxylic acid cycle, exerting a negative effect on recombinant protein biosynthesis (Celik et al. 2010). Another critical aspect of exponential methanol feeding strategies is the potential risk of localized oxygen limitations, even when the dissolved oxygen level in the medium is >30%. Accelerated cell growth under such conditions is associated with increased oxygen demand, which particularly in high-density cultures, may lead to an undersupply of oxygen (Heyland et al. 2011) and results in downregulated methanol assimilation pathways (Yu et al. 2022). Moreover, a 25% increase in oxygen consumption was observed throughout fermentation under the exponential feeding strategy (Fig. S5), along with increased heat generation, which contributed to higher operational costs than those of the limited strategy. Additionally, elevated methanol concentrations can lead to toxic effects. A study reported that methanol levels exceeding 5% (v/v) inhibited cell growth (Wakayama et al. 2016). Although *K.* *phaffii* can tolerate these methanol concentrations, toxic effects can occur, particularly under conditions of limited oxygen availability (Wakayama et al. 2016). These findings show that exponential feeding strategies accelerate cell growth. However, they also increase the risk of metabolic burden, which can reduce the yield of the target protein.

The collagen-based protein concentrations measured in this study are unusually high compared with values reported in the literature. Studies involving the expression of similar human collagen proteins in *K.* *phaffii* have reported final titers ranging from 0.5 g L⁻^1^ to 16 g L⁻^1^ (Xiang et al. 2022; Nokelainen et al. 2001; Ma et al. 2022; Liu et al. 2011). This discrepancy is attributed primarily to the quantification method used in this study, which relies on ELISA-based detection of a C-terminal His-tag. Establishing a precise and reliable quantification approach has proven challenging; therefore, this assay was chosen as a practical compromise. Since the target protein was not commercially available, a purified reference isolated by affinity chromatography was used as the standard for quantification. While the absolute protein concentrations quantified by ELISA are not directly comparable to published data, the use of the same analytical method across all experimental conditions allows a relative evaluation of the investigated process parameters. Nevertheless, it should be noted that the ELISA detection method may overestimate the actual protein content. To improve the reliability and interpretability of future studies, the integration of alternative, high-resolution analytical techniques, such as chromatography or mass spectrometry, should be considered.

The implementation of a three-phase fermentation strategy, with an additional glycerol fed-batch following the initial batch growth phase, was intended to increase the cell density at the beginning of methanol induction to increase recombinant protein expression. As expected, the extended growth period resulted in a higher cell density of approx. 200 g L⁻^1^ WCW. However, none of the approaches had a positive effect on the final ColMP-His concentration. This has been reported in other studies, where initiating induction at lower cell densities resulted in higher yields of the target protein (Jia et al. 2013, 2017; Wang et al. 2012). In particular, under limiting methanol feeding conditions, the final protein yield was reduced with the additional glycerol fed-batch phase. The ColMP-His concentration reached only 22.6 g L⁻^1^, which was 43% of the protein obtained without prior glycerol supplementation. In addition, high cell densities, accompanied by cellular stress, can promote the accumulation of extracellular proteases in the culture media. These enzymes degrade recombinant proteins and represent a critical factor contributing to product loss (Schlauch et al. 2024; Gellermann et al. 2019; Werten et al. 1999). One study demonstrated that protease activity can be reduced up to 88% by initiating induction at low cell densities in combination with controlled cell growth. This approach minimizes metabolic stress by preventing oxygen limitation and enhancing methanol utilization, resulting in increased cell viability, reduced extracellular protease release, and improved recombinant protein expression (Wang et al. 2012). The additional growth phase also contributes to increased operational costs owing to extended fermentation process times and an observed 14% increase in oxygen consumption (Fig. S4). This negatively impacts the cost-efficiency of the production process because no increased product yield was observed, which would justify the additional costs.

The present data indicate that maximizing cell density is not a major factor for efficient recombinant protein production for ColMP-His. Rather, controlled cell growth under physiologically optimal conditions appears to be critical for achieving high protein productivity, which has also been demonstrated in previous studies (Boojari et al. 2023; Zhang et al. 2000). To further optimize the process, various methanol feed rates were evaluated, including a gradient-based strategy with stepwise increased feed. This strategy resulted in the highest final product concentration (83.9 g L⁻^1^) and was the only condition that resulted in a continuous increase in protein titer and biomass throughout the entire fermentation period. This observation highlights the balance between cell growth and recombinant protein expression. In contrast to constant feed rates, an adaptive control strategy allows for gradual metabolic adjustment, thereby minimizing the risk of overfeeding during the early induction phase and reducing the limitations of underfeeding at later stages. This approach reduces cellular stress and promotes sustained protein secretion over extended periods by dynamically adjusting methanol availability to meet specific metabolic demands at each stage of fermentation.

Although high variability in the measured protein concentration and a limited number of biological replicates did not allow statistical confirmation, the observed trends consistently favored the gradient-based strategy, underscoring its potential for further optimizations and scale-up applications.

## Conclusion

This study investigated different strategies for the optimization of methanol feeding in a *K.* *phaffii* expression system with respect to their suitability for efficient production of a recombinant collagen-mimetic protein. The results demonstrate that neither maximum biomass accumulation nor increased exponential methanol feed necessarily correlates with higher product yields. Instead, a methanol-limited process strategy without a preceding glycerol fed-batch growth phase enabled the highest protein production. The gradient-based methanol feeding strategy supported continuous protein accumulation throughout the entire fermentation process, thereby representing a promising foundation for the cost-efficient manufacturing of collagen-based raw materials. For large-scale use, future efforts should focus on improving batch-to-batch reproducibility, integrating process analytical technologies, and advancing methods for protein quantification. The described strategies provide a foundation for scalable bioprocesses to produce animal-free biomaterials, offering promising contributions to the development of functional bioinks for applications in tissue engineering and bioprinting technologies.

## Supplementary Information

Below is the link to the electronic supplementary material.

ESM1 (DOCX 2.65 MB)

## Data Availability

Data is provided within the manuscript or supplementary information files.

## References

[CR1] Boojari MA, Rajabi Ghaledari F, Motamedian E, Soleimani M, Shojaosadati SA (2023) Developing a metabolic model-based fed-batch feeding strategy for *Pichia pastoris* fermentation through fine-tuning of the methanol utilization pathway. Microb Biotechnol 16:1344–1359. 10.1111/1751-7915.14264 37093126 10.1111/1751-7915.14264 PMC10221529

[CR2] Brierley RA (1998) Secretion of recombinant human insulin-like growth factor I (IGF-I). Methods Mol Biol 103:149–177. 10.1385/0-89603-421-6:149 9680639 10.1385/0-89603-421-6:149

[CR3] Butkowski RJ, Noelken ME, Hudson BG (1982) Estimation of the size of collagenous proteins by electrophoresis and gel chromatography. Methods in Enzymology : Structural and Contractile Proteins Part A: Extracellular Matrix. Academic Press, pp 410–423

[CR4] Cai H-L, Doi R, Shimada M, Hayakawa T, Nakagawa T (2021) Metabolic regulation adapting to high methanol environment in the methylotrophic yeast *Ogataea methanolica*. Microb Biotechnol 14:1512–1524. 10.1111/1751-7915.13811 33939325 10.1111/1751-7915.13811 PMC8313246

[CR5] Cao L, Zhang Z, Yuan D, Yu M, Min J (2024) Tissue engineering applications of recombinant human collagen: a review of recent progress. Front Bioeng Biotechnol 12:1358246. 10.3389/fbioe.2024.1358246 38419725 10.3389/fbioe.2024.1358246 PMC10900516

[CR6] Celik E, Calik P, Halloran SM, Oliver SG (2007) Production of recombinant human erythropoietin from *Pichia pastoris* and its structural analysis. J Appl Microbiol 103:2084–2094. 10.1111/j.1365-2672.2007.03448.x 18045392 10.1111/j.1365-2672.2007.03448.x

[CR7] Celik E, Calik P, Oliver SG (2009) Fed-batch methanol feeding strategy for recombinant protein production by *Pichia pastoris* in the presence of co-substrate sorbitol. Yeast 26:473–484. 10.1002/yea.1679 19575480 10.1002/yea.1679

[CR8] Celik E, Calik P, Oliver SG (2010) Metabolic flux analysis for recombinant protein production by *Pichia pastoris* using dual carbon sources: effects of methanol feeding rate. Biotechnol Bioeng 105:317–329. 10.1002/bit.22543 19777584 10.1002/bit.22543

[CR9] Chen Z, Fan D, Shang L (2020) Exploring the potential of the recombinant human collagens for biomedical and clinical applications: a short review. Biomed Mater 16:12001. 10.1088/1748-605X/aba6fa 10.1088/1748-605X/aba6fa 32679570

[CR10] Chen J, Fan Y, Dong G, Zhou H, Du R, Tang X, Ying Y, Li J (2023) Designing biomimetic scaffolds for skin tissue engineering. Biomater Sci 11:3051–3076. 10.1039/d3bm00046j 36970875 10.1039/d3bm00046j

[CR11] Chiruvolu V, Cregg JM, Meagher MM (1997) Recombinant protein production in an alcohol oxidase-defective strain of *Pichia pastoris* in fedbatch fermentations. Enzyme Microb Technol 21:277–283. 10.1016/S0141-0229(97)00042-2

[CR12] Cregg JM, Cereghino JL, Shi J, Higgins DR (2000) Recombinant protein expression in *Pichia pastoris*. Mol Biotechnol 16:23–52. 10.1385/MB:16:1:23 11098467 10.1385/MB:16:1:23

[CR13] De S, Mattanovich D, Ferrer P, Gasser B (2021) Established tools and emerging trends for the production of recombinant proteins and metabolites in *Pichia pastoris*. Essays Biochem 65:293–307. 10.1042/EBC20200138 33956085 10.1042/EBC20200138

[CR14] Dietzsch C, Spadiut O, Herwig C (2011) A dynamic method based on the specific substrate uptake rate to set up a feeding strategy for *Pichia pastoris*. Microb Cell Fact 10:14. 10.1186/1475-2859-10-14 21371310 10.1186/1475-2859-10-14 PMC3059269

[CR15] Friess W (1998) Collagen—biomaterial for drug delivery. Eur J Pharm Biopharm 45:113–136. 10.1016/S0939-6411(98)00017-4 9704909 10.1016/s0939-6411(98)00017-4

[CR16] Fukuda K, Hori H, Utani A, Burbelo PD, Yamada Y (1997) Formation of recombinant triple-helical alpha 1(IV)2 alpha 2(IV) collagen molecules in CHO cells. Biochem Biophys Res Commun 231:178–182. 10.1006/bbrc.1997.6069 9070244 10.1006/bbrc.1997.6069

[CR17] Furthmayr H, Timpl R (1971) Characterization of collagen peptides by sodium dodecylsulfate-polyacrylamide electrophoresis. Anal Biochem 41:510–516. 10.1016/0003-2697(71)90173-4 4326447 10.1016/0003-2697(71)90173-4

[CR18] Garrigós-Martínez J, Vuoristo K, Nieto-Taype MA, Tähtiharju J, Uusitalo J, Tukiainen P, Schmid C, Tolstorukov I, Madden K, Penttilä M, Montesinos-Seguí JL, Valero F, Glieder A, Garcia-Ortega X (2021) Bioprocess performance analysis of novel methanol-independent promoters for recombinant protein production with *Pichia pastoris*. Microb Cell Fact 20:74. 10.1186/s12934-021-01564-9 33757505 10.1186/s12934-021-01564-9 PMC7986505

[CR19] Geddis AE, Prockop DJ (1993) Expression of human COL1A1 gene in stably transfected HT1080 cells: the production of a thermostable homotrimer of type I collagen in a recombinant system. Matrix 13:399–405. 10.1016/S0934-8832(11)80045-4 8246835 10.1016/s0934-8832(11)80045-4

[CR20] Gellermann P, Schneider-Barthold C, Bolten SN, Overfelt E, Scheper T, Pepelanova I (2019) Production of a recombinant non-hydroxylated gelatin mimetic in *Pichia pastoris* for biomedical applications. J Funct Biomater 10:39. 10.3390/jfb10030039 31480684 10.3390/jfb10030039 PMC6787575

[CR21] Haagdorens M, Edin E, Fagerholm P, Groleau M, Shtein Z, Ulčinas A, Yaari A, Samanta A, Cepla V, Liszka A, Tassignon M-J, Simpson F, Shoseyov O, Valiokas R, Pintelon I, Ljunggren MK, Griffith M (2022) Plant recombinant human collagen type I hydrogels for corneal regeneration. Regen Eng Transl Med 8:269–283. 10.1007/s40883-021-00220-3

[CR22] Hayashi T, Nagai Y (1980) The anomalous behavior of collagen peptides on sodium dodecyl sulfate-polyacrylamide gel electrophoresis is due to the low content of hydrophobic amino acid residues. J Biochem 87:803–808 7390962 10.1093/oxfordjournals.jbchem.a132809

[CR23] Heyland J, Fu J, Blank LM, Schmid A (2011) Carbon metabolism limits recombinant protein production in *Pichia pastoris*. Biotechnol Bioeng 108:1942–1953. 10.1002/bit.23114 21351072 10.1002/bit.23114

[CR24] Jia D, Liu L, Wang H, Zhang D, Li J, Du G, Chen J (2013) Overproduction of a truncated poly (vinyl alcohol) dehydrogenase in recombinant *Pichia pastoris* by low-temperature induction strategy and related mechanism analysis. Bioprocess Biosyst Eng 36:1095–1103. 10.1007/s00449-012-0863-5 23207825 10.1007/s00449-012-0863-5

[CR25] Jia L, Tu T, Huai Q, Sun J, Chen S, Li X, Shi Z, Ding J (2017) Enhancing monellin production by *Pichia pastoris* at low cell induction concentration via effectively regulating methanol metabolism patterns and energy utilization efficiency. PLoS ONE 12:e0184602. 10.1371/journal.pone.0184602 28981536 10.1371/journal.pone.0184602 PMC5628809

[CR26] Jordà J, Jouhten P, Cámara E, Maaheimo H, Albiol J, Ferrer P (2012) Metabolic flux profiling of recombinant protein secreting *Pichia pastoris* growing on glucose:methanol mixtures. Microb Cell Fact 11:57. 10.1186/1475-2859-11-57 22569166 10.1186/1475-2859-11-57 PMC3443025

[CR27] Karbalaei M, Rezaee SA, Farsiani H (2020) *Pichia pastoris*: a highly successful expression system for optimal synthesis of heterologous proteins. J Cell Physiol 235:5867–5881. 10.1002/jcp.29583 32057111 10.1002/jcp.29583 PMC7228273

[CR28] Liu B, Lei YT, Zhang J, Hu L, Yang SL (2011) Expression, purification and characterization of recombinant human gelatin in *Pichia pastoris*. Antimicrob Agents Chemother 236:2905–2912. 10.4028/www.scientific.net/AMR.236-238.2905

[CR29] Ma XX, Fan DD, Zhu CH, Shang ZF, Mi Y (2014) Optimization of fermentation medium for collagen production of recombinant *Pichia pastoris* during induction phase. J Chem Pharm Res 6:1802–1809

[CR30] Ma L, Liang X, Yu S, Zhou J (2022) Expression, characterization, and application potentiality evaluation of recombinant human-like collagen in *Pichia pastoris*. Bioresour Bioprocess 9:119. 10.1186/s40643-022-00606-3 38647896 10.1186/s40643-022-00606-3 PMC10992492

[CR31] Merrett K, Wan F, Lee C-J, Harden JL (2021) Enhanced collagen-like protein for facile biomaterial fabrication. ACS Biomater Sci Eng 7:1414–1427. 10.1021/acsbiomaterials.1c00069 33733733 10.1021/acsbiomaterials.1c00069

[CR32] Moser JW, Prielhofer R, Gerner SM, Graf AB, Wilson IBH, Mattanovich D, Dragosits M (2017) Implications of evolutionary engineering for growth and recombinant protein production in methanol-based growth media in the yeast *Pichia pastoris*. Microb Cell Fact 16:49. 10.1186/s12934-017-0661-5 28302114 10.1186/s12934-017-0661-5 PMC5356285

[CR33] Moura Campos S, dos Santos Costa G, Karp SG, Thomaz-Soccol V, Soccol CR (2025) Innovations and challenges in collagen and gelatin production through precision fermentation. World J Microbiol Biotechnol 41:63. 10.1007/s11274-025-04276-z 39910024 10.1007/s11274-025-04276-z

[CR34] Myllyharju J, Lamberg A, Notbohm H, Fietzek PP, Pihlajaniemi T, Kivirikko KI (1997) Expression of wild-type and modified proalpha chains of human type I procollagen in insect cells leads to the formation of stable alpha1(I)2alpha2(I) collagen heterotrimers and alpha1(I)3 homotrimers but not alpha2(I)3 homotrimers. J Biol Chem 272:21824–21830. 10.1074/jbc.272.35.21824 9268313 10.1074/jbc.272.35.21824

[CR35] Myllyharju J, Nokelainen M, Vuorela A, Kivirikko KI (2000) Expression of recombinant human type I-III collagens in the yeast *Pichia pastoris*. Biochem Soc Trans 28:353–357. 10.1042/bst0280353 10961918

[CR36] Nokelainen M (2000) Recombinant human collagens: characterization of type II collagen expressed in insect cells and production of types I-III collagen in the yeast *Pichia pastoris. *Dissertation, University of Oulu

[CR37] Nokelainen M, Tu H, Vuorela A, Notbohm H, Kivirikko KI, Myllyharju J (2001) High-level production of human type I collagen in the yeast *Pichia pastoris*. Yeast 18:797–806. 10.1002/yea.730 11427962 10.1002/yea.730

[CR38] Qi Q, Yao L, Liang Z, Yan D, Li Z, Huang Y, Sun J (2016) Production of human type II collagen using an efficient baculovirus-silkworm multigene expression system. Mol Genet Genomics 291:2189–2198. 10.1007/s00438-016-1251-7 27669694 10.1007/s00438-016-1251-7

[CR39] Rutschmann C, Baumann S, Cabalzar J, Luther KB, Hennet T (2014) Recombinant expression of hydroxylated human collagen in *Escherichia coli*. Appl Microbiol Biotechnol 98:4445–4455. 10.1007/s00253-013-5447-z 24362857 10.1007/s00253-013-5447-z

[CR40] Schlauch D, Ebbecke JP, Meyer J, Fleischhammer TM, Pirmahboub H, Kloke L, Kara S, Lavrentieva A, Pepelanova I (2024) Development of a human recombinant collagen for vat polymerization-based bioprinting. Biotechnol J 19:e202400393. 10.1002/biot.202400393 39380502 10.1002/biot.202400393

[CR41] Shoseyov O, Posen Y, Grynspan F (2014) Human collagen produced in plants: more than just another molecule. Bioengineered 5:49–52. 10.4161/bioe.26002 23941988 10.4161/bioe.26002 PMC4008466

[CR42] Stein H, Wilensky M, Tsafrir Y, Rosenthal M, Amir R, Avraham T, Ofir K, Dgany O, Yayon A, Shoseyov O (2009) Production of bioactive, post-translationally modified, heterotrimeric, human recombinant type-I collagen in transgenic tobacco. Biomacromol 10:2640–2645. 10.1021/bm900571b 10.1021/bm900571b 19678700

[CR43] Stratton J, Chiruvolu V, Meagher M (1998) High cell-density fermentation. Methods Mol Biol 103:107–120. 10.1385/0-89603-421-6:107 9680637 10.1385/0-89603-421-6:107

[CR44] Wakayama K, Yamaguchi S, Takeuchi A, Mizumura T, Ozawa S, Tomizuka N, Hayakawa T, Nakagawa T (2016) Regulation of intracellular formaldehyde toxicity during methanol metabolism of the methylotrophic yeast *Pichia methanolica*. J Biosci Bioeng 122:545–549. 10.1016/j.jbiosc.2016.03.022 27094957 10.1016/j.jbiosc.2016.03.022

[CR45] Wang H, Li J, Liu L, Li X, Jia D, Du G, Chen J, Song J (2012) Increased production of alkaline polygalacturonate lyase in the recombinant *Pichia pastoris* by controlling cell concentration during continuous culture. Bioresour Technol 124:338–346. 10.1016/j.biortech.2012.08.027 22995164 10.1016/j.biortech.2012.08.027

[CR46] Wang S, Wang Y, Yuan Q, Yang L, Zhao F, Lin Y, Han S (2023) Development of high methanol-tolerance *Pichia pastoris* based on iterative adaptive laboratory evolution. Green Chem 25:8845–8857. 10.1039/D3GC02874G

[CR47] Wang C, Guo X, Fan M, Yue L, Wang H, Wang J, Zha Z, Yin H (2024) Production of recombinant human type I collagen homotrimers in CHO cells and their physicochemical and functional properties. J Biotechnol 395:149–160. 10.1016/j.jbiotec.2024.09.011 39357624 10.1016/j.jbiotec.2024.09.011

[CR48] Werten MWT, van den Bosch TJ, Wind RD, Mooibroek H, de Wolf FA (1999) High-yield secretion of recombinant gelatins by *Pichia pastoris*. Yeast 15:1087–1096. 10.1002/(SICI)1097-0061(199908)15:11<1087:AID-YEA436>3.0.CO;2-F 10.1002/(SICI)1097-0061(199908)15:11<1087::AID-YEA436>3.0.CO;2-F 10455232

[CR49] Werten MW, Wisselink WH, van den Jansen- Bosch TJ, de Bruin EC, de Wolf FA (2001) Secreted production of a custom-designed, highly hydrophilic gelatin in *Pichia pastoris*. Protein Eng 14:447–454. 10.1093/protein/14.6.447 11477225 10.1093/protein/14.6.447

[CR50] Williams KE, Olsen DR (2021) Gelatin expression from an engineered *Saccharomyces cerevisiae* CUP1 promoter in *Pichia pastoris*. Yeast 38:382–387. 10.1002/yea.3554 33580598 10.1002/yea.3554

[CR51] Xiang Z-X, Gong J-S, Shi J-H, Liu C-F, Li H, Su C, Jiang M, Xu Z-H, Shi J-S (2022) High-efficiency secretory expression and characterization of the recombinant type III human-like collagen in *Pichia pastoris*. Bioresour Bioprocess 9:117. 10.1186/s40643-022-00605-4 38647563 10.1186/s40643-022-00605-4 PMC10992891

[CR52] Xie W, Wu Q, Kuang Z, Cong J, Zhang Q, Huang Y, Su Z, Xiang Q (2023) Temperature-controlled expression of a recombinant human-like collagen I peptide in *Escherichia coli*. Bioengineering 10:926. 10.3390/bioengineering10080926 37627811 10.3390/bioengineering10080926 PMC10451535

[CR53] Xu X, Gan Q, Clough RC, Pappu KM, Howard JA, Baez JA, Wang K (2011) Hydroxylation of recombinant human collagen type I alpha 1 in transgenic maize co-expressed with a recombinant human prolyl 4-hydroxylase. BMC Biotechnol 11:69. 10.1186/1472-6750-11-69 21702901 10.1186/1472-6750-11-69 PMC3151215

[CR54] Yu Y-F, Yang J, Zhao F, Lin Y, Han S (2022) Comparative transcriptome and metabolome analyses reveal the methanol dissimilation pathway of *Pichia pastoris*. BMC Genomics 23:366. 10.1186/s12864-022-08592-8 35549850 10.1186/s12864-022-08592-8 PMC9103059

[CR55] Zahrl RJ, Peña DA, Mattanovich D, Gasser B (2017) Systems biotechnology for protein production in *Pichia pastoris*. FEMS Yeast Res 17(7). 10.1093/femsyr/fox068 10.1093/femsyr/fox068 28934418

[CR56] Zhang W, Inan M, Meagher MM (2000) Fermentation strategies for recombinant protein expression in the methylotrophic yeast *Pichia pastoris*. Biotechnol Bioprocess Eng 5:275–287. 10.1007/BF02942184

