# Supplementary material for: Methanol feeding strategies for high-yield production of a collagen-based protein in Komagataella phaffii
Source: Appl Microbiol Biotechnol. 2025 Dec 24;109(1):284. doi: 10.1007/s00253-025-13675-z (PMC12740954; doi:10.1007/s00253-025-13675-z)
Supplement: Supplementary file 1 — (DOCX 2.65 MB) [file 253_2025_13675_MOESM1_ESM.docx]

Supplementary Information

Methanol Feeding Strategies for High-Yield Production of a Collagen-Based Protein in *Komagataella phaffii*

in Applied Microbiology and Biotechnology

Jan Peter Ebbecke^1,2^, Domenic Schlauch^1,2^, Charlotte Güler^1,2^, Hamidreza Pirmahboub^2^, Selin Kara^1^, Iliyana Pepelanova^1^

Correspondence: Jan Peter Ebbecke (ebbecke@iftc.uni-hannover.de)

^1^ Institute of Technical Chemistry, Leibniz University Hannover, Hannover, Germany

^2^ Cellbricks GmbH, Berlin, Germany

Table of contents

[1 Methods for SDS-PAGE and Western Blot 1](#_Toc206840807)

[1.1 Characterization of protein expression with (SDS-PAGE) 1](#_Toc206840808)

[1.2 Identification of expressed target protein by Western Blot 1](#_Toc206840809)

[2 SDS-PAGE results 1](#_Toc206840810)

[2.1 SDS-PAGE: Protein secretion during fermentation for comparison limited vs exponential methanol feeding strategy 1](#_Toc206840811)

[2.2 SDS-PAGE: Protein secretion during fermentation for impact of additional glycerol fed batch 1](#_Toc206840812)

[2.3 SDS-PAGE: Protein secretion during fermentation for comparison of different methanol feeding rates 2](#_Toc206840813)

[3 Specific ColMP-His expressions rate according to Wet cell weight 2](#_Toc206840814)

[4 Bioprocess data: DO, Oxygen supplementation and Oxygen consumption total (lim vs. exp) 3](#_Toc206840815)

[5 Significance Analysis of the Impact of an adjusted methanol feed rate on cell growth and target protein expression 3](#_Toc206840816)

[6 Specific growth rate of two-phases and three-phases fermentations with limited and exponential methanol feeding strategy 5](#_Toc206840817)

[7 Western Blot for validate the ColMP-His molecular weight analysis 6](#_Toc206840818)

[8 Comprehensive summary of the process parameters and yields from performed fermentations 7](#_Toc206840819)

1. Methods for SDS-PAGE and Western Blot
   1. Characterization of protein expression with (SDS-PAGE)

For the SDS-PAGE analysis, the sample was collected from the reactor vessel and centrifuged at 13,000 rpm for 10 minutes at 15 °C. Supernatant containing the target protein was filtered through a 0.2 µm membrane filter and stored at -20 °C for further use. Entire fermentation samples were thawed immediately prior to analysis, mixed with 4x Laemmli buffer and incubated for 6 minutes at 96 °C. 8 µL of sample were loaded onto a 12 % gel. A pre-stained protein ladder (Thermo Fisher Scientific, Cat. 26616) was used for size comparison. After electrophoresis, the gels were incubated in demineralized water followed by protein staining with Coomassie Brilliant Blue G250 for visualization.

- 1. Identification of expressed target protein by Western Blot

For Western blot analysis, sample preparation was performed as described in Section 1.1. Following gel electrophoresis, proteins were transferred onto a PVDF membrane (Bio-Rad Laboratories) at 15 V for 50 minutes. The membrane was subsequently blocked using skim milk powder. Detection of His₆-tagged proteins was carried out using a horseradish peroxidase (HRP)-conjugated anti-His₆ antibody (Invitrogen) in combination with Pierce 1-Step Ultra TMB blotting solution (Thermo Fisher Scientific).

1. SDS-PAGE results
   1. SDS-PAGE: Protein secretion during fermentation for comparison limited vs exponential methanol feeding strategy


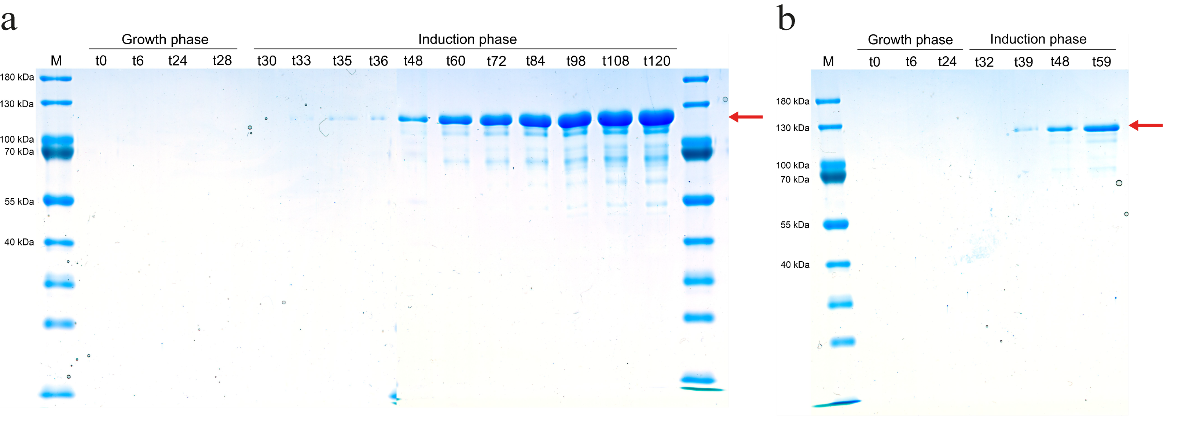


**Fig. S1** SDS-PAGE from the ColMP-His expression of limited methanol feed (a) and exponential methanol feed (b) without additional fed-batch growth phase, samples of the cell-free culture supernatant taken during the fermentation are shown.

- 1. SDS-PAGE: Protein secretion during fermentation for impact of additional glycerol fed batch


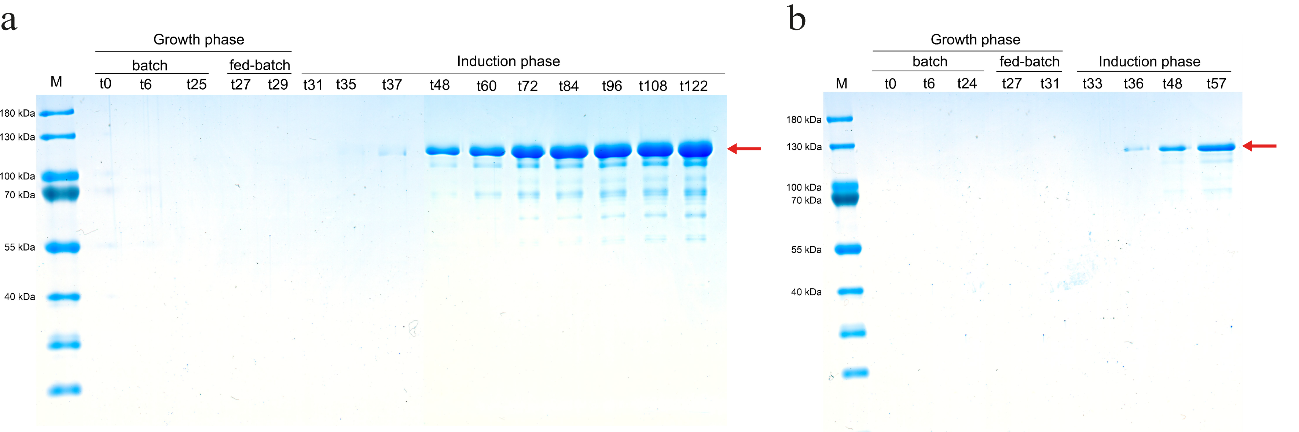


**Fig. S2** SDS-PAGE from the ColMP-His expression of limited methanol feed (a) and exponential methanol feed (b) with additional fed-batch growth phase, samples of the cell-free culture supernatant taken during the fermentation are shown

- 1. SDS-PAGE: Protein secretion during fermentation for comparison of different methanol feeding rates


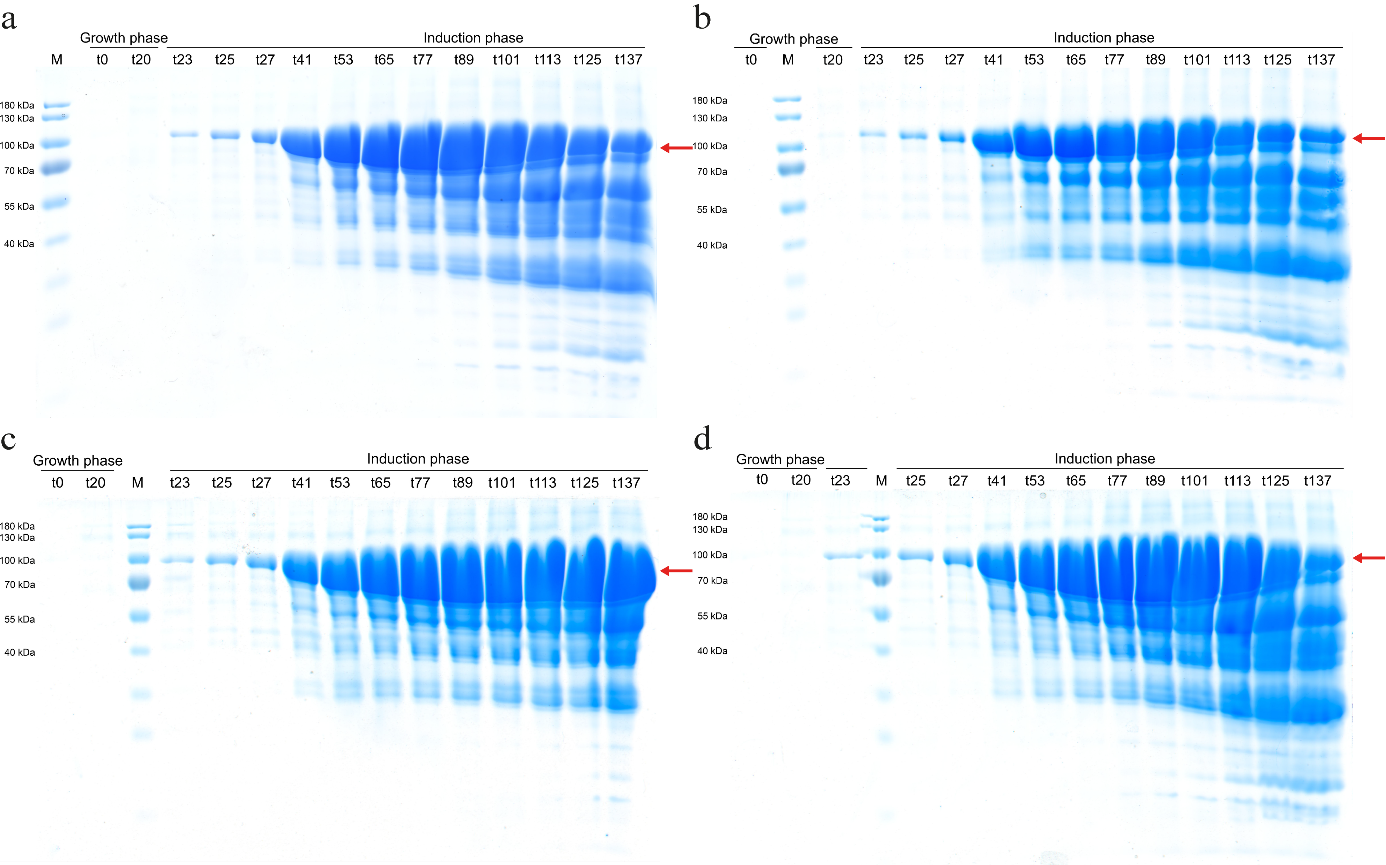


**Fig. S3** SDS-PAGE from the ColMP-His expression of limited feed with standard methanol flow (a), increased methanol flow (b), decreased methanol flow (c) and stepwise increased gradient methanol flow (d), samples of the cell-free culture supernatant taken during the fermentation are shown

1. Specific ColMP-His expressions rate according to Wet cell weight


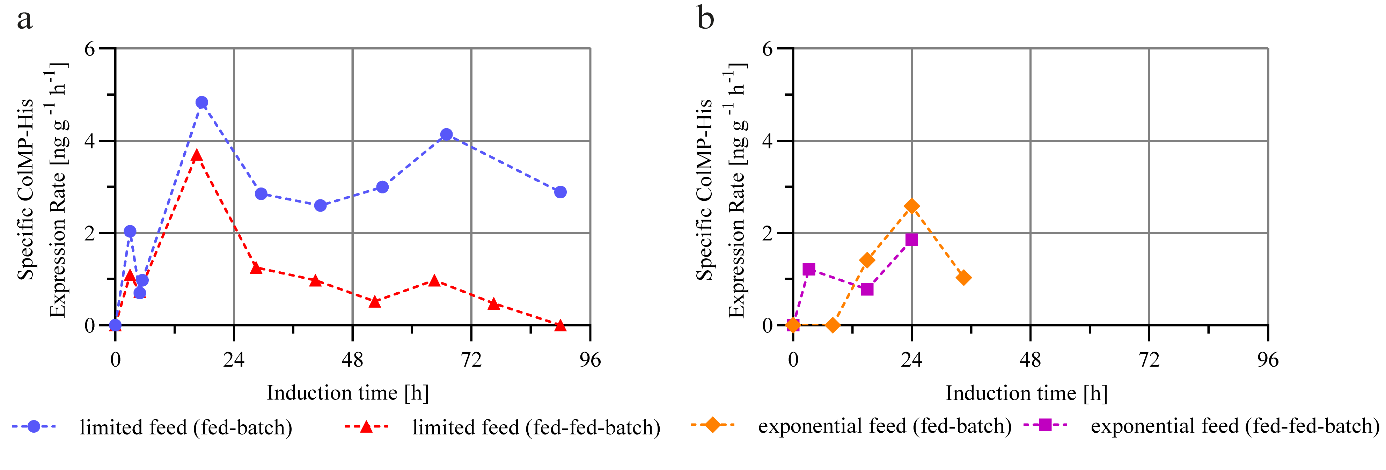


**Fig. S4** Specific ColMP-His expressions rate according to Wet cell weight (WCW) [ng g^-1^] during the induction phase of fermentation processes with an additional glycerol fed-batch phase (fed-fed-batch, red/violet) and without (fed-batch, blue/orange). Two different methanol feeding strategies were investigated: (a) limited feed (constant methanol flow into culture medium) and (b) exponential feed (constant methanol concentration in the culture medium)

1. Bioprocess data: DO, Oxygen supplementation and Oxygen consumption total (lim vs. exp)


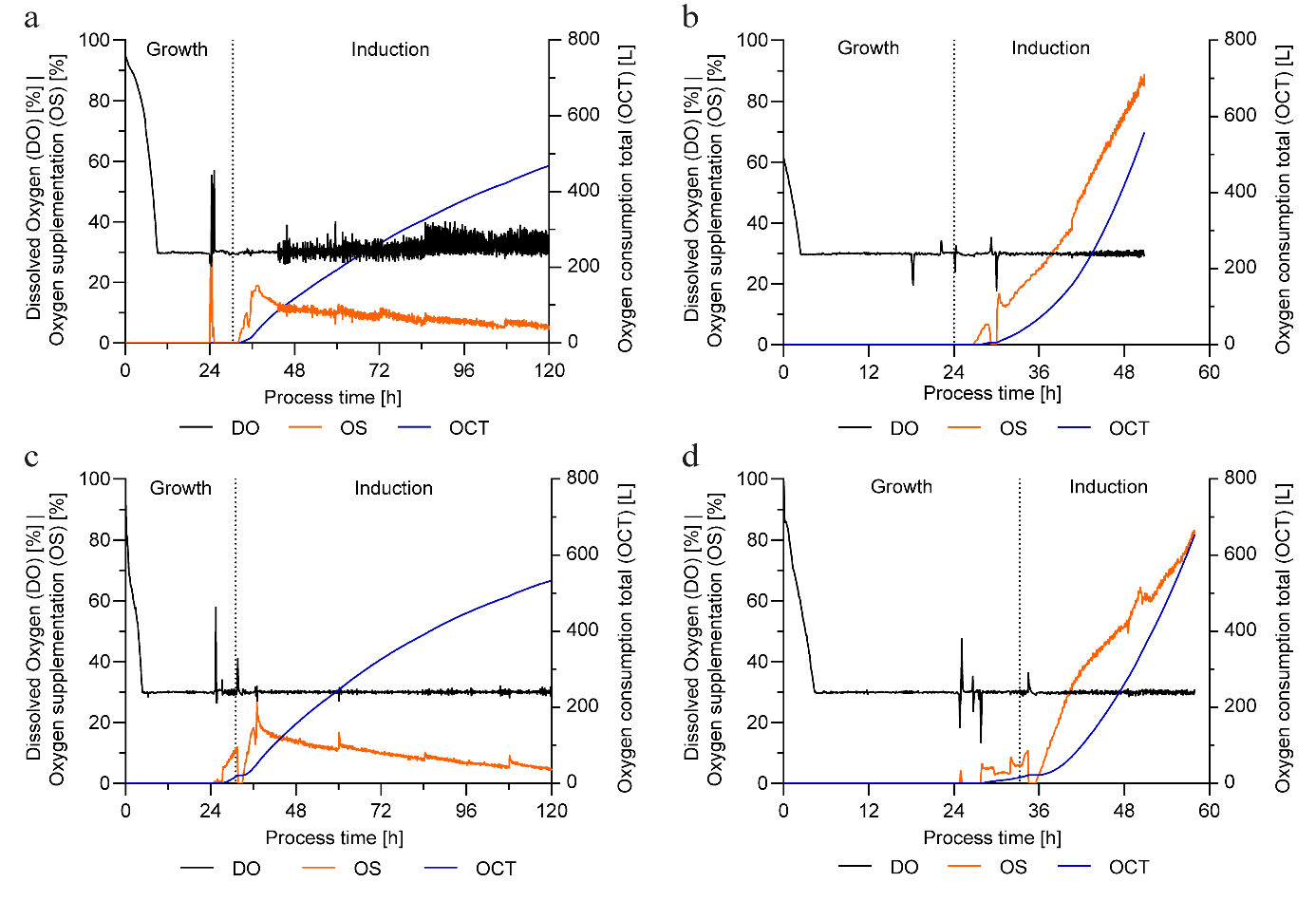


**Fig. S5** Online Bioprocess data of Dissolved Oxygen (DO) [%], Oxygen supplementation (OS) [%] and Oxygen consumption total (OCT) [L] according process time [h] of fermentations under two different methanol feeding strategies with and without additional glycerol fed-batch phase: (a) limited feed (constant methanol flow into culture medium) without glycerol fed-batch, (c) with glycerol fed-batch and (b) exponential feed (constant methanol concentration in the culture medium) without glycerol fed-batch, (d) with glycerol fed-batch

1. Significance Analysis of the Impact of an adjusted methanol feed rate on cell growth and target protein expression

In the previous investigations, differences in cell growth and recombinant protein expression were observed as a function of the applied methanol feeding strategy. The variant with a stepwise-increasing methanol feed rate resulted in the highest cell density and the highest ColMP-His concentration throughout the induction phase. To statistically evaluate these results, the fermentation processes with standard and gradient-based methanol feed rates were performed in independent triplicates. For the significance analysis, the respective sampling points of WCW and ColMP-His concentration throughout the induction period were considered. Data evaluation was conducted using an unpaired t-test, with a significance level of p  <  0.05 defined as the threshold for identifying statistically significant differences. The presented results are based on arithmetic means, accompanied by the respective standard deviations. Sampling was conducted at regular intervals between 0 h and 117 h of induction to adequately capture the temporal progression of the investigated parameters.


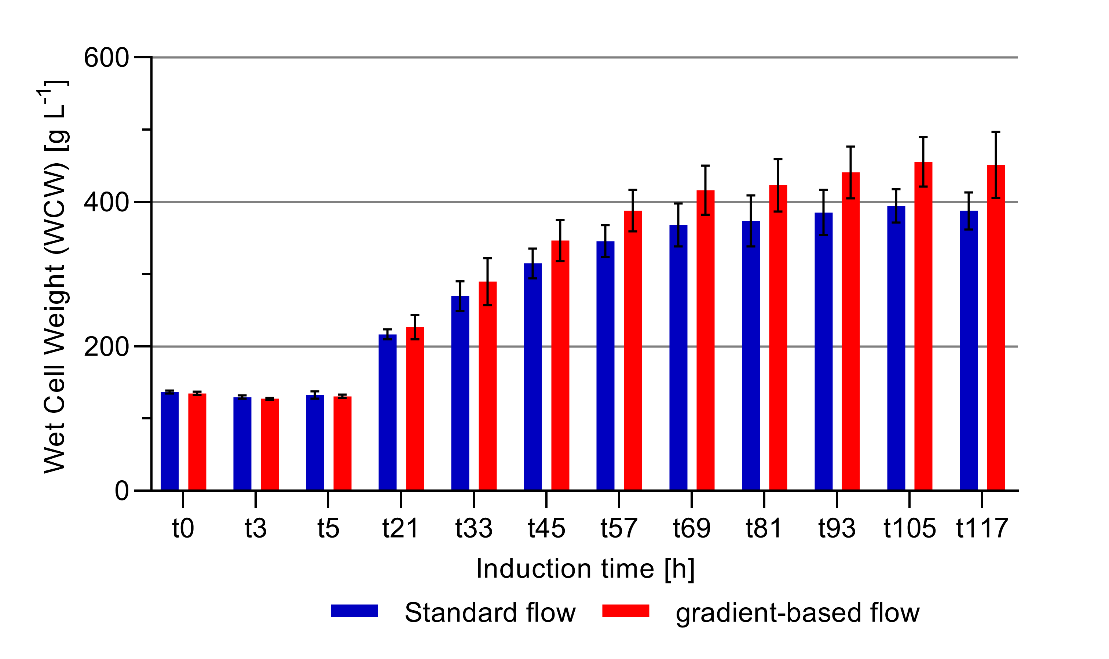


**Fig. S6** Wet cell weight (WCW) [g L^-1^] throughout the induction phase of fermentation processes with standard (10,9 mL h^-1^ L^-1^_initial vol._ ) and gradient-based (10,9 mL h^-1^ L^-1^_initial vol._+1 mL h^-1^ L^-1^_initial vol._ every 12h) methanol feeding rate. Data represents mean values from fermentation triplicates, including standard deviation

Both methanol feeding strategies showed a continuous increase in cell mass following the adaptation phase, beginning at 5 h of induction (Fig. S6). Cell growth was particularly pronounced between 5 h and 60 h and began to slow after 80 h, consistent with the typical transition into the stationary phase. From 20 h to 30 h of induction, the WCW under the gradient-based feeding strategy consistently exceeded that of the standard strategy. This difference continued to increase throughout the induction period, resulting in a mean final difference of 64 g L^-1^ between the two conditions at the last sample taken. Subsequent statistical analysis using an unpaired t-test (p < 0.05) revealed that no statistically significant differences in WCW were observed between these two methanol feeding strategies at any sampling point.


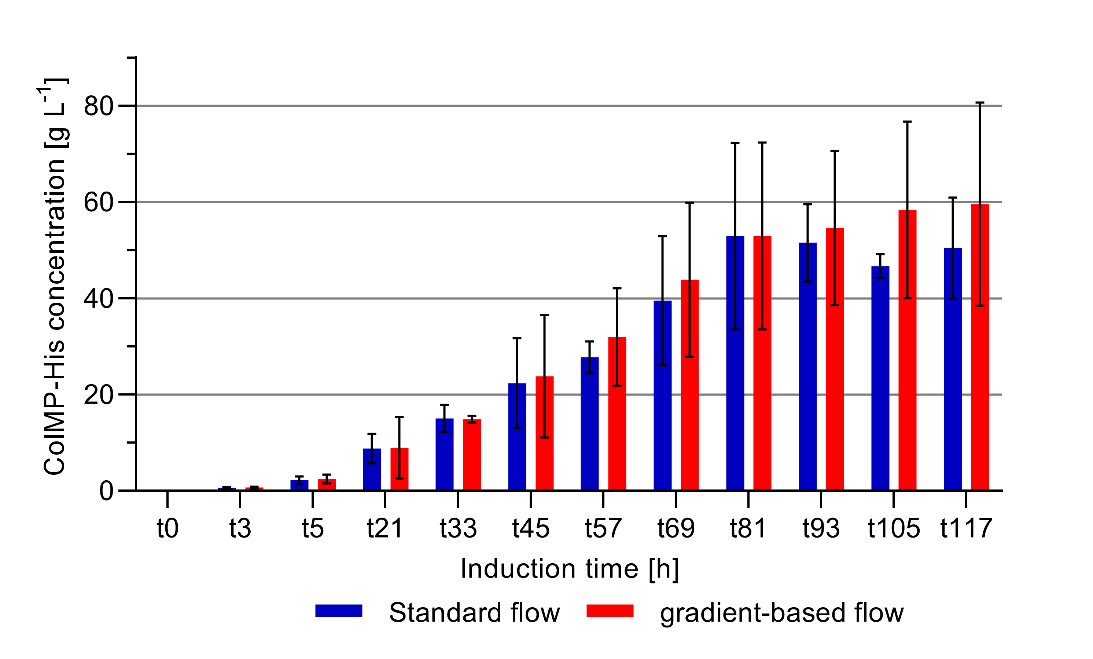


**Fig. S7** ColMP-His concentration in cell-free culture supernatant [g L^-1^] throughout the induction phase of fermentation processes with standard (10,9 mL h^-1^ L^-1^_initial vol._ ) and gradient-based (10,9 mL h^-1^ L^-1^_initial vol._+1 mL h^-1^ L^-1^_initial vol._ every 12h) methanol feeding rate. Data represents mean values from fermentation triplicates, including standard deviation

In correlation with the observed cell growth, protein concentration increased continuously in both methanol feeding strategies following completion of the methanol adaptation phase (Fig. S7). In both cases, a steady rise in ColMP-His concentration was observed up to approximately 81 h of induction. During this period, no marked differences between the two strategies were apparent at any sampling point.

Subsequently, the standard flow fermentation exhibited a stable to slightly declining protein level, reaching 50 g L^-1^ ColMP-His by end of induction phase. In contrast, gradient-based fermentation showed a continued increase in protein concentration during the same interval, ultimately reaching a final titer of 59.6 g L^-1^. This representing an 19 % higher protein yield compared to the standard strategy.

Despite this visible difference, statistical analysis of the data did not reveal any significant differences in ColMP-His concentration between these two strategies at any sampling point. This result can largely be attributed to high standard deviations and the limited number of fermentation replicates. In particular, during the interval from 81 h to 117 h, relative standard deviations of up to 36.5 % were observed, which considerably impaired the reliability of statistical interpretation.

To enable statistically robust conclusions in future studies, optimization of the protein quantification method and an increase in statistical power through additional replicates will be essential.

1. Specific growth rate of two-phases and three-phases fermentations with limited and exponential methanol feeding strategy


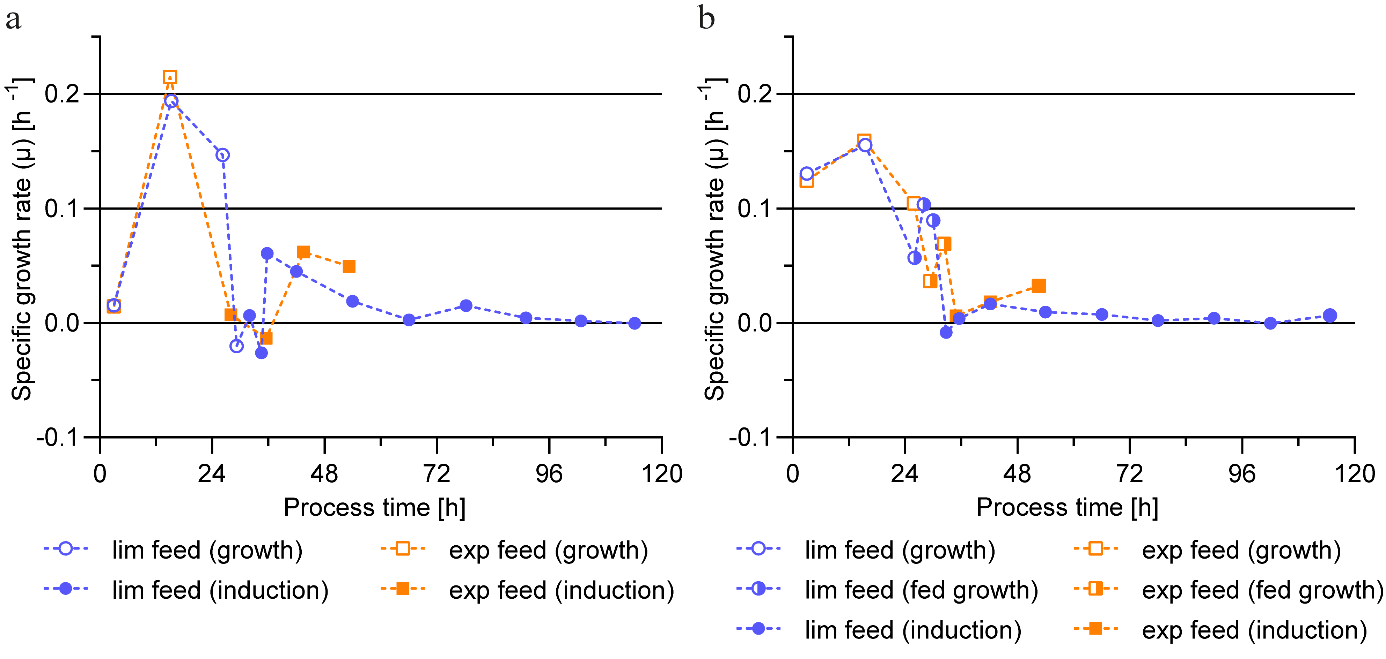


**Fig. S8** Specific growth rate (µ) [h^-1^] of two-phases (a) and three-phases fermentations (b) with limited (blue) and exponential (orange) methanol feeding strategy. The three-phase fermentation was performed with an additional glycerol-limited fed-batch phase. The glycerol batch phase (unfilled markers), the glycerol-limited fed-batch phase (half-filled markers) and the methanol induction phase (filled markers) are shown.

1. Western Blot for validate the ColMP-His molecular weight analysis


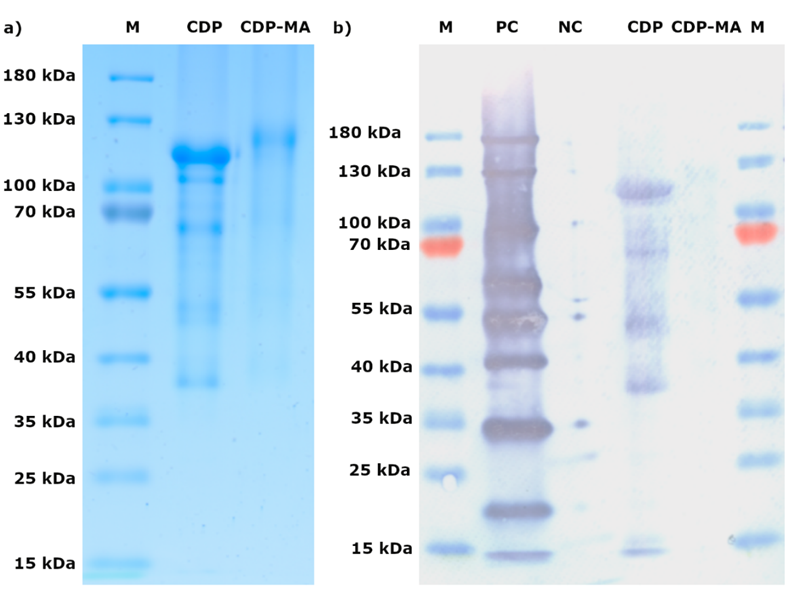


**Fig. S9** SDS-PAGE of the unfunctionalized CDP (=ColMP-His) and CDP-MA (=functionalized ColMP-His). b) Western blot of CDP and CDP-MA with His-tagged protein ladder as positive control (PC) and bovine serum albumin as negative control (NC) (Schlauch et al. 2024).

1. Comprehensive summary of the process parameters and yields from performed fermentations

**Tab. S1** Comprehensive summary of the process parameters and yields from performed fermentations with respect to the comparison between the two-phase and three-phase fermentation strategies as well as the differences between the limiting and exponential methanol-feed strategies.

|  | Two-phases fermentation | | Three-phases fermentation | |
| --- | --- | --- | --- | --- |
| Methanol-feed strategy | limited | exponential | limited | exponential |
| Methanol | 10.9  [mL h^-1^ L^-1^] _in. vol._ | 0,5% (v/v) | 10.9  [mL h^-1^ L^-1^] _in. vol._ | 0,5% (v/v) |
| Fermentation media volume [L] | 1 | 1 | 1 | 1 |
|  |  |  |  |  |
| Phase duration [h]: |  |  |  |  |
| Growth _batch_ | 30.5 | 24 | 25 | 27 |
| Growth _fed-batch_ | - | - | 6 | 6 |
| Induction _fed-batch_ | 90 | 34.5 | 90 | 24 |
|  |  |  |  |  |
| Consumption: |  |  |  |  |
| Glycerol (S) [g L^-1^] | 60 | 60 | 147 | 147 |
| Glycerol [g L^-1^ h^-1^] | - | - | 6 | 6 |
| Methanol (S) [mL L^-1^] | 1000 | 600 | 100 | 880 |
| Methanol [mL L^-1^ h^-1^] | 11.1 | 17.4 | 11.1 | 36.7 |
|  |  |  |  |  |
| Yields: |  |  |  |  |
| Biomasse _end growth_ (X) [g L^-1^] | 115 | 115 | 200 | 202 |
| Biomasse _end induction_ (X) [g L^-1^] | 331 | 326 | 342 | 343 |
| ColMP-His conc. (P) [g L^-1^] | 51.3 | 7.5 | 22.4 | 8.0 |
|  |  |  |  |  |
| Cell yield _on Glycerol_ (Y_X/S_) [g g^-1^] | 1.917 | 1.917 | 1.361 | 1.374 |
| Cell yield _on Methanol_ (Y_X/S_) [g mL^-1^] | 0.216 | 0.352 | 0.142 | 0.160 |
| Protein _yield on Methanol_ (Y_P/S_) [g mL^-1^] | 0.051 | 0.013 | 0220. | 0.009 |
| Space-Time-Yield (Q_P_) [g L^-1^ h^-1^] | 0.426 | 0.128 | 0.185 | 0.140 |

**Tab. S2** Comprehensive summary of the process parameters and yields from performed fermentations with respect to the influence of the methanol feed rate under a limited methanol-feed in the two-phases fermentation strategy.

|  | Two-phases fermentation | | | |
| --- | --- | --- | --- | --- |
| Methanol-feed strategy | limited | | | |
| Methanol-feed rate | standard | increased | decreased | Gradient-based |
| [mL h^-1^ L^-1^] _initial volume_ | 10.9 | 14.5 | 7.3 | 10.9  + 1 (every 12h) |
| Fermentation media volume [L] | 0.7 | 0.7 | 0.7 | 0.7 |
|  |  |  |  |  |
| Phase duration [h]: |  |  |  |  |
| Growth _batch_ | 20 | 20 | 20 | 20 |
| Induction _fed-batch_ | 117 | 117 | 117 | 117 |
|  |  |  |  |  |
| Consumption: |  |  |  |  |
| Glycerol (S) [g L^-1^] | 60 | 60 | 60 | 60 |
| Glycerol [g L^-1^ h^-1^] | 3.0 | 3.0 | 3.0 | 3.0 |
| Methanol (S) [mL L^-1^] | 1275 | 1722 | 880 | 1814 |
| Methanol [mL L^-1^ h^-1^] | 10.9 | 14.7 | 7.3 | 15.5 |
|  |  |  |  |  |
| Yields: |  |  |  |  |
| Biomasse _end growth_ (X) [g L^-1^] | 135 | 136 | 132 | 132 |
| Biomasse _end induction_ (X) [g L^-1^] | 405 | 457 | 384 | 500 |
| ColMP-His conc. (P) [g L^-1^] | 62.5 | 56.0 | 60.0 | 83.9 |
|  |  |  |  |  |
| Cell yield _on Glycerol_ (Y_X/S_) [g g^-1^] | 2.250 | 2.267 | 2.200 | 2.200 |
| Cell yield _on Methanol_ (Y_X/S_) [g mL^-1^] | 0.212 | 0.186 | 0.286 | 0.203 |
| Protein _yield on Methanol_ (Y_P/S_) [g mL^-1^] | 0.049 | 0.033 | 0.068 | 0.046 |
| Space-Time-Yield (Q_P_) [g L^-1^ h^-1^] | 0.456 | 0.409 | 0.438 | 0.612 |
